# *Polygonum cognatum* Extract: Multitarget Anti‐inflammatory, Antidiabetic, and Epigenetic Modulation Properties

**DOI:** 10.1002/open.202500505

**Published:** 2026-02-04

**Authors:** Serhat Karaman, Yakup Budak, Elif Aktürk Bozdemir

**Affiliations:** ^1^ Faculty of Medicine Department of Emergency Medicine Tokat Gaziosmanpasa University Tokat Turkiye; ^2^ Faculty of Arts and Sciences Department of Chemistry Tokat Gaziosmanpasa University Tokat Turkiye; ^3^ Tokat Vocational School Tokat Gaziosmanpasa University Tokat Turkiye

**Keywords:** anti‐inflammatory, antidiabetic, LC‐MS/MS, molecular docking, multitarget bioactivity, phenolic compounds, *Polygonum cognatum*

## Abstract

*Polygonum cognatum* (Madımak) is a plant traditionally consumed for medicinal purposes in Turkey. Unlike previous studies examining samples from different regions and seasons, this research presents the first comprehensive characterization of *P. cognatum* collected from the Central Black Sea Region (Tokat, 40°01′02″N, 36°28′15″E; 1210 m altitude) during the vegetative growth phase (June 2024), where geographical origin and collection time significantly influence secondary metabolite profiles. This study evaluates the phytochemical profile and multitarget biological activities of *P. cognatum* extracts obtained using solvents of different polarities (hexane, ethanol, and water). Advanced analytical techniques (liquid chromatography–tandem mass spectrometry, high‐performance liquid chromatography‐diode array detector, and gas chromatography‐mass spectrometry) identified 28 phenolic compounds, with the ethanol extract showing the highest diversity (24 compounds) and total phenolic content (78.6 ± 2.3 mg GAE/g). Compounds identified for the first time in *P. cognatum* include isoquercetin‐3‐O‐rhamnoside, apigenin‐7‐O‐glucoside, and luteolin‐4′‐O‐glucoside. The ethanol extract demonstrated superior multitarget bioactivity: potent antioxidant activity ( 2,2‐diphenyl‐1‐picrylhydrazyl (DPPH) IC_50_: 76.4 ± 2.1 μg/mL), **moderate but selective** anti‐inflammatory effects (COX‐2 IC_50_: 145.3 ± 5.2 μg/mL; selectivity index: 2.06, indicating preferential COX‐2 inhibition over COX‐1) and significant antidiabetic potential (*α*‐amylase IC_50_: 89.3 ± 3.1 μg/mL; *α*‐glucosidase IC_50_: 76.8 ± 2.9 μg/mL), and antimicrobial activity (MIC: 62.5 μg/mL against *S. aureus*). Notably, this study demonstrates for the first time the histone deacetylase (HDAC) inhibitory activity of *P. cognatum* (IC_50_: 92.4 ± 3.8 μg/mL), revealing novel epigenetic modulation properties. Molecular docking studies showed strong correlations between binding affinities and experimental IC_50_ values (*r* = −0.87 to −0.91; *p* < 0.01). Cytotoxicity evaluation showed favorable safety profiles (CC_50_ > 500 μg/mL). Docking, IC_50_, and compositional data consistently indicate that quercetin, rutin, chlorogenic acid, and kaempferol are key contributors to the observed antioxidant, antidiabetic, anti‐inflammatory, and HDAC inhibitory effects. These findings establish *P. cognatum* as a promising multitarget therapeutic agent with novel epigenetic regulatory mechanisms, supporting its potential development for inflammatory, metabolic, and epigenetic‐related disorders.

## Graphical abstract

*Polygonum cognatum* extracts exhibit strong antioxidant, anti‐inflammatory, antidiabetic, and antimicrobial activities. This study demonstrates, for the first time, histone deacetylase inhibitory activity, revealing epigenetic modulation potential. A rich phenolic profile, including quercetin, kaempferol, and chlorogenic acid, underlies these multitarget effects. Molecular docking analyses show strong correlation with experimental data, providing mechanistic insight.”

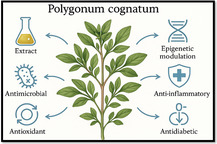

## 1 Introduction

Medicinal and aromatic plants have served as therapeutic agents for centuries, with their bioactive compounds offering alternatives to synthetic pharmaceuticals that increasingly face challenges from side effects and antimicrobial resistance [1, 2]. According to the World Health Organization, ≈80% of the global population relies on herbal products for primary healthcare, necessitating a systematic investigation of the phytochemical content and biological activities of medicinal plants [3].

The genus *Polygonum* (Polygonaceae) comprises ≈300 phytochemically rich species worldwide, containing a diverse array of bioactive compounds, including flavonoids, anthraquinones, terpenoids, and phenolic compounds [4, 5, 6]. These species are extensively utilized in traditional medicine systems across Asia and Europe for their anti‐inflammatory, antimicrobial, antioxidant, antidiabetic, and anticancer properties [7, 8]. Recent advances in analytical chemistry, particularly liquid chromatography–tandem mass spectrometry (LC‐MS/MS) techniques, have enabled the precise identification and quantification of bioactive compounds, facilitating structure–activity relationship studies and mechanistic investigations [9, 10].

*Polygonum cognatum* (Madımak), endemic to Turkey's Central and Eastern Anatolia regions, serves dual purposes as both a traditional food and medicinal plant [11]. Traditional applications include the treatment of inflammatory conditions, microbial infections, diabetes, and skin disorders, while its young shoots are consumed as nutrient‐rich vegetables [12, 13, 14, 15]. Furthermore, the increasing prevalence of metabolic disorders such as diabetes and inflammatory diseases has intensified the search for natural compounds with multitarget therapeutic potential [16, 17].

Phenolic‐rich plant matrices have been increasingly recognized as promising multitarget therapeutic candidates, with dual anti‐inflammatory and antiglycation activities, and detailed phytochemical profiling reported for related medicinal taxa [18, 19]. Despite its traditional significance, the comprehensive phytochemical and biological characterization of *P. cognatum* itself remains limited. Previous studies have examined individual extracts: methanol extracts have shown potent radical scavenging activity [13], antimicrobial efficacy has been demonstrated particularly against Gram‐positive bacteria [12], and in vivo antidiabetic effects have been reported [20]. However, no systematic comparative analysis of *P. cognatum* extracts obtained with solvents of different polarity has been conducted, representing a significant knowledge gap.

Solvent polarity has a critical influence on extraction efficiency and the bioactive compound profiles. Polar solvents (water, ethanol) effectively extract phenolic acids and flavonoids, while apolar solvents (hexane) preferentially isolate terpenoids and fatty acids [21, 22, 23]. This selectivity directly impacts the biological activity spectrum of the resulting extracts [21, 24]. Modern drug discovery emphasizes multitarget therapeutics that simultaneously address multiple pathological pathways, a characteristic naturally possessed by plant extracts due to their complex phytochemical compositions [25]. Computational approaches, such as molecular docking studies, have become invaluable for understanding the molecular mechanisms underlying biological activities, enabling the prediction of drug‐target interactions and facilitating rational phytopharmaceutical design [26, 27].

Integrating advanced analytical techniques (LC‐MS/MS), comprehensive bioactivity screening (antioxidant, antimicrobial, anti‐inflammatory, and antidiabetic), and computational studies provides a holistic approach to medicinal plant research, validating traditional uses while identifying new therapeutic applications [28, 29].

Recent advances in epigenetic research have highlighted the importance of histone modifications in regulating gene expression and cellular function. Histone deacetylases (HDACs) remove acetyl groups from histones, leading to chromatin condensation and transcriptional repression [30]. HDAC inhibitors have emerged as promising therapeutic agents for various diseases, including cancer, neurodegenerative disorders, and inflammatory conditions [31]. Natural products have shown significant potential as HDAC inhibitors with reduced toxicity compared to synthetic compounds [32]. However, the epigenetic modulation properties of Polygonum species, including *P. cognatum*, remain largely unexplored, representing a significant knowledge gap in understanding their comprehensive therapeutic potential.

We hypothesize that *P. cognatum* extracts obtained using different polarity solvents will exhibit distinct phytochemical profiles and bioactivities, with phenolic compounds serving as the primary bioactive constituents responsible for their multitarget therapeutic effects.

This study aims to provide a comprehensive characterization of *P. cognatum* through: (1) advanced phytochemical profiling using LC‐MS/MS analysis, (2) multitarget bioactivity assessment including antioxidant, antimicrobial, anti‐inflammatory, antidiabetic, and cytotoxic properties, (3) molecular docking studies to elucidate mechanisms of action, and (4) structure–activity relationship analysis. This research establishes a scientific foundation for *P. cognatum*'s therapeutic potential, supporting its development as a standardized phytopharmaceutical for multitarget applications in modern medicine.

## 2 Results

### 2.1 Yield of Extracts

The yields of *P. cognatum* extracts obtained with solvents of different polarities are presented in Table 1. The highest yield was observed for the ethanol extract (12.8% ± 0.5%), while the lowest yield was observed for the hexane extract (3.2% ± 0.2%). The yield of the extracts was directly proportional to the solvent polarity (hexane < water < ethanol). This indicates that *P. cognatum* is richer in polar compounds. Similar results have been reported for other members of the *Polygonum* species [32, 33].

**TABLE 1:** Yield and phytochemical content of extracts of *P. cognatum* obtained with solvents of different polarities.

Extract	Yield (%)	Phenolic content (mg GAE/g)	Flavonoid content (mg QE/g)
Hexane	3.2 ± 0.2^c^	12.4 ± 0.8^c^	8.6 ± 0.5^c^
Ethanol	12.8 ± 0.5^a^	78.6 ± 2.3^a^	42.3 ± 1.8^a^
Water	9.5 ± 0.4^b^	45.2 ± 1.5^b^	23.7 ± 1.2^b^

Values are expressed as mean ± standard deviation (*n* = 3). Different letters (a, b, c) in the same column indicate significant differences between groups (*p* < 0.05, Tukey HSD test).

### 2.2 Phytochemical Content

The total phenolic and flavonoid contents of *P. cognatum* extracts are presented in Table 1. Ethanol extract had the highest total phenolic (78.6 ± 2.3 mg GAE/g) and flavonoid (42.3 ± 1.8 mg QE/g) contents. This was followed by water and hexane extracts, respectively. These results indicate that ethanol is the most suitable solvent for extracting phenolic compounds and flavonoids.

### 2.3 LC‐MS/MS Analysis

A total of 28 phenolic compounds were identified and quantified in *P. cognatum* extracts (Table 2). The ethanol extract showed the highest diversity with 24 compounds, followed by the water extract (18 compounds) and the hexane extract (8 compounds). Compounds reported for the first time in this species include: isoquercetin‐3‐O‐rhamnoside: isoquercetin‐3‐O‐rhamnoside (2.3 ± 0.1 mg/g), apigenin‐7‐O‐glucoside (1.8 ± 0.1 mg/g), and luteolin‐4′‐O‐glucoside (1.5 ± 0.1 mg/g) in ethanol extract.

**TABLE 2:** Phenolic compounds identified by LC‐MS/MS in *P. cognatum* extracts (Major compounds).

Compound	RT (min)	[M‐H]^−^ (m/z)	Hexane (mg/g)	Ethanol (mg/g)	Water (mg/g)	LOD	LOQ	*R* ^2^
Gallic acid	3.2	169	2.1 ± 0.1^c^	15.4 ± 0.8^a^	12.3 ± 0.6^b^	0.05	0.15	0.998
Protocatechuic acid	5.8	153	1.8 ± 0.1^c^	8.7 ± 0.4^a^	6.2 ± 0.3^b^	0.08	0.25	0.997
Hydroxybenzoic acid	6.4	137	1.2 ± 0.1^c^	5.3 ± 0.3^a^	3.8 ± 0.2^b^	0.06	0.18	0.996
Catechin	8.4	289	3.2 ± 0.2^c^	12.6 ± 0.7^a^	9.8 ± 0.5^b^	0.12	0.35	0.996
Vanillic acid	9.7	167	0.9 ± 0.1^c^	4.2 ± 0.2^a^	3.1 ± 0.2^b^	0.04	0.12	0.999
Syringic acid	11.3	197	1.1 ± 0.1^c^	3.8 ± 0.2^a^	2.9 ± 0.1^b^	0.05	0.16	0.998
Chlorogenic acid	12.1	353	4.5 ± 0.3^c^	18.9 ± 1.1^a^	14.2 ± 0.8^b^	0.10	0.30	0.999
Caffeic acid	14.7	179	2.8 ± 0.2^c^	11.3 ± 0.6^a^	8.7 ± 0.4^b^	0.07	0.20	0.998
Epicatechin	16.3	289	3.1 ± 0.2^c^	10.8 ± 0.6^a^	7.9 ± 0.4^b^	0.11	0.32	0.997
p‐Coumaric acid	18.9	163	1.9 ± 0.1^c^	7.4 ± 0.4^a^	5.8 ± 0.3^b^	0.06	0.18	0.999
Ferulic acid	21.5	193	2.3 ± 0.1^c^	9.2 ± 0.5^a^	6.7 ± 0.4^b^	0.09	0.28	0.998
Sinapic acid	22.8	223	1.4 ± 0.1^c^	5.6 ± 0.3^a^	4.2 ± 0.2^b^	0.07	0.22	0.997
Rutin	24.8	609	5.8 ± 0.4^c^	22.7 ± 1.3^a^	18.4 ± 1.0^b^	0.15	0.45	0.996
Hyperoside	26.1	463	2.7 ± 0.2^c^	8.9 ± 0.5^a^	6.8 ± 0.4^b^	0.12	0.36	0.998
Isoquercitrin	27.4	463	3.1 ± 0.2^c^	9.7 ± 0.6^a^	7.3 ± 0.4^b^	0.13	0.38	0.997
Quercetin	28.3	301	4.2 ± 0.3^c^	16.8 ± 0.9^a^	12.1 ± 0.7^b^	0.13	0.40	0.997
Naringenin	29.7	271	1.8 ± 0.1^c^	6.4 ± 0.4^a^	4.7 ± 0.3^b^	0.09	0.27	0.996
Kaempferol	31.7	285	3.6 ± 0.2^c^	14.5 ± 0.8^a^	10.3 ± 0.6^b^	0.12	0.38	0.998
Apigenin	33.2	269	2.1 ± 0.1^c^	7.8 ± 0.4^a^	5.9 ± 0.3^b^	0.10	0.31	0.997
Luteolin	34.5	285	1.9 ± 0.1^c^	6.9 ± 0.4^a^	5.2 ± 0.3^b^	0.11	0.33	0.998
Chrysin	36.8	253	1.3 ± 0.1^c^	4.7 ± 0.3^a^	3.4 ± 0.2^b^	0.08	0.25	0.996
Myricetin	25.9	317	2.4 ± 0.2^c^	8.1 ± 0.5^a^	6.2 ± 0.3^b^	0.12	0.37	0.997
Ellagic acid	19.8	301	1.7 ± 0.1^c^	5.9 ± 0.3^a^	4.5 ± 0.3^b^	0.09	0.28	0.998
Resveratrol	23.4	227	0.8 ± 0.1^c^	3.2 ± 0.2^a^	2.4 ± 0.1^b^	0.05	0.16	0.999
Procyanidin B1	10.7	577	2.9 ± 0.2^c^	9.4 ± 0.6^a^	7.1 ± 0.4^b^	0.14	0.42	0.996
Procyanidin B2	13.8	577	2.6 ± 0.2^c^	8.7 ± 0.5^a^	6.5 ± 0.4^b^	0.13	0.40	0.997
Epicatechin gallate	20.3	441	1.5 ± 0.1^c^	5.8 ± 0.3^a^	4.3 ± 0.2^b^	0.11	0.34	0.998
Quercetin‐3‐glucoside	26.7	463	2.8 ± 0.2^c^	9.2 ± 0.5^a^	7.0 ± 0.4^b^	0.12	0.37	0.997

RT: Retention time; values are expressed as mean ± standard deviation (*n* = 3). Different letters (a, b, c) in the same row indicate significant differences between groups (*p* < 0.05). LOD/LOQ values in μg/mL. Compound identification was confirmed by comparing the retention times (with a tolerance of ±0.1 min) and mass spectra of the samples with those of authentic standards, all of which were analyzed under identical chromatographic conditions.

### 2.4 Antimicrobial Activity

The antimicrobial activity of *P. cognatum* extracts was evaluated using the disk diffusion and microdilution methods. The results are presented in Tables 3 and 4.

**TABLE 3:** Antimicrobial activity of *P. cognatum* extracts (zone of inhibition, mm).

Test microorganism	Hexane extract	Ethanol extract	Water extract	Gentamicin	Fluconazole
**Bacteria**					
*S. aureus* ATCC 25923	12.4 ± 0.5^b^	18.2 ± 0.7^a^	10.5 ± 0.4^c^	22.6 ± 0.8	—
*B. subtilis* ATCC 6633	10.8 ± 0.4^b^	16.5 ± 0.6^a^	9.2 ± 0.3^c^	20.3 ± 0.7	—
*E. coli* ATCC 25922	8.5 ± 0.3^b^	12.7 ± 0.5^a^	7.3 ± 0.3^c^	18.5 ± 0.6	—
*P. aeruginosa* ATCC 27853	6.2 ± 0.2^b^	9.8 ± 0.4^a^	5.4 ± 0.2^c^	16.2 ± 0.5	—
*S. typhimurium* ATCC 14028	7.8 ± 0.3^b^	11.5 ± 0.5^a^	6.8 ± 0.3^c^	17.4 ± 0.6	—
**Fungi**					
*C. albicans* ATCC 10231	9.5 ± 0.4^b^	14.2 ± 0.6^a^	8.3 ± 0.3^c^	—	18.7 ± 0.7
*A. niger* ATCC 16404	7.2 ± 0.3^b^	10.8 ± 0.4^a^	6.5 ± 0.2^c^	—	15.3 ± 0.5

Values are expressed as mean ± standard deviation (*n* = 3). Different letters (a, b, c) in the same row indicate significant differences between groups (*p* < 0.05, Tukey's HSD test).

**TABLE 4:** MIC (μg/mL) of *P. cognatum* extracts.

Test microorganism	Hexane extract	Ethanol extract	Water extract	Gentamicin	Fluconazole
**Bacteria**					
*S. aureus* ATCC 25923	125	62.5	250	0.5	—
*B. subtilis* ATCC 6633	250	125	500	1	—
*E. coli* ATCC 25922	500	250	1000	2	—
*P. aeruginosa* ATCC 27853	>1000	500	>1000	4	—
*S. typhimurium* ATCC 14028	500	250	1000	2	—
**Fungi**					
*C. albicans* ATCC 10231	250	125	500	—	2
*A. niger* ATCC 16404	500	250	1000	—	4

### 2.5 Anti‐inflammatory and Antidiabetic Activities

The anti‐inflammatory and antidiabetic activities of *P. cognatum* extracts are presented in Table 5. These results demonstrate the multitarget therapeutic potential of *P. cognatum*, extending beyond its traditional antimicrobial and antioxidant applications.

**TABLE 5:** Anti‐inflammatory and antidiabetic activities of *P. cognatum* extracts.

Extract/Control	COX‐1 IC_50_ (μg/mL)	COX‐2 IC_50_ (μg/mL)	Selectivity index	*α*‐Amylase IC_50_ (μg/mL)	*α*‐Glucosidase IC_50_ (μg/mL)	Protein denaturation inhibition (%)
Hexane	485.2 ± 15.3	267.8 ± 9.1^a^	1.81	156.7 ± 5.8^a^	142.3 ± 4.9^a^	45.3 ± 2.1
Ethanol	298.7 ± 8.1	145.3 ± 5.2^c^	2.06	**89.3 ± 3.1^c^**	**76.8 ± 2.9^c^**	78.4 ± 3.2
Water	378.9 ± 12.4	198.6 ± 7.3^b^	1.91	125.4 ± 4.6^b^	108.2 ± 3.7^b^	62.1 ± 2.8
Acarbose	—	—	—	85.2 ± 2.8^d^	73.1 ± 2.5^d^	—

Values are expressed as mean ± standard deviation (*n* = 3). Statistical analysis was performed using one‐way ANOVA followed by Tukey's HSD post‐hoc test. *p* < 0.05 was considered statistically significant.

The ethanol extract exhibited significant COX‐2 selectivity, with IC_50_ values of 145.3 ± 5.2 μg/mL (COX‐2) and 298.7 ± 8.1 μg/mL (COX‐1), resulting in a selectivity index of 2.06. The extract also showed 78.4% ± 3.2% inhibition of protein denaturation at a concentration of 500 μg/mL. The ethanol extract exhibited potent antidiabetic activity, with IC_50_ values of 89.3 ± 3.1 μg/mL for *α*‐amylase inhibition and 76.8 ± 2.9 μg/mL for *α*‐glucosidase inhibition, comparable to acarbose (IC_50_ values of 85.2 ± 2.8 and 73.1 ± 2.5 μg/mL, respectively).

### 2.6 HDAC Inhibitory Activity

The HDAC inhibitory activity of *P. cognatum* extracts was evaluated to investigate their potential epigenetic modulation properties. The results are presented in Table 6.

**TABLE 6:** HDAC inhibitory activity of *P. cognatum* extracts.

Extract/Control	HDAC IC_50_ (μg/mL)
Hexane	>500^a^
Ethanol	92.4 ± 3.8^c^
Water	178.6 ± 6.5^b^
Vorinostat (SAHA)	2.1 ± 0.2 (μM)^d^

Values are expressed as mean ± standard deviation (*n* = 3). Statistical analysis was performed using one‐way ANOVA followed by Tukey's HSD post‐hoc test. *p* < 0.05 was considered statistically significant.

**FIGURE 1:**
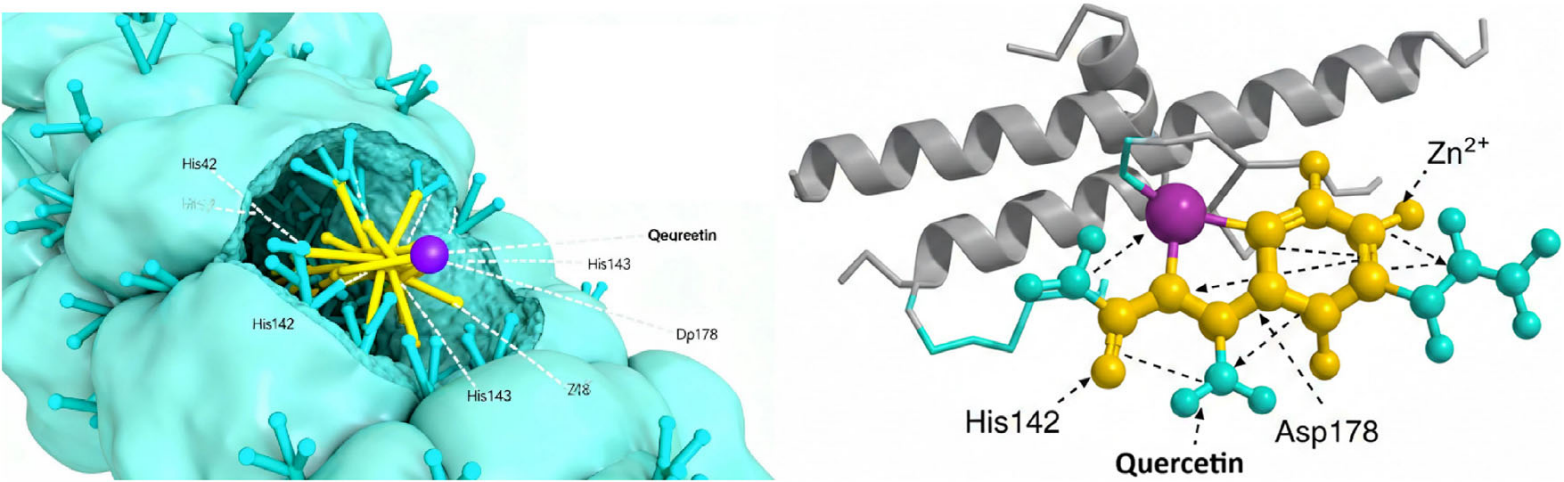
Molecular docking simulation of quercetin binding to the HDAC8 active site.

The ethanol extract demonstrated significant HDAC inhibitory activity with an IC_50_ value of 92.4 ± 3.8 μg/mL, followed by the water extract (178.6 ± 6.5 μg/mL). The hexane extract exhibited minimal activity (IC_50_ > 500 μg/mL). These results correlate with the phenolic content of the extracts, suggesting that phenolic compounds may be responsible for the observed HDAC inhibitory activity. Previous studies have reported that flavonoids, particularly quercetin and kaempferol, possess HDAC inhibitory properties through interactions with the zinc‐binding domain of HDAC enzymes [34]. Molecular docking analysis further confirmed the binding interactions of quercetin within the catalytic pocket of HDAC8, highlighting coordination with Zn^2+^ and hydrogen bonding with His142, His143, and Asp178 residues (Figure 1).

### 2.7 Molecular Docking Studies

To elucidate the molecular mechanisms underlying the observed biological activities, molecular docking studies were performed to investigate the binding interactions of major phenolic compounds identified in *P. cognatum* extracts with key target enzymes. The docking results are presented in Table 7.

**TABLE 7:** Molecular docking results of major phenolic compounds with target enzymes.

Compound	COX‐2 binding affinity (kcal/mol)	Key interactions (COX‐2)	* α*‐Amylase binding affinity (kcal/mol)	Key interactions (*α*‐Amylase)	* α*‐Glucosidase binding affinity (kcal/mol)	Key interactions (*α*‐Glucosidase)
Quercetin	−9.2	Arg120, Tyr355, Phe518	−7.8	Asp197, Glu233	−8.9	Asp214, Asp349
Kaempferol	−8.8	Arg120, Tyr355	−7.5	Asp197, Asp300	−8.6	Asp214, Arg442
Chlorogenic acid	−8.1	Arg120, Ser530	−8.7	Asp197, Glu233, Asp300	−8.3	Asp214, Asp349
Rutin	−8.9	Arg120, Tyr355, Ser530	−8.2	Asp197, Glu233	−9.5	Asp214, Asp349, Arg442

The molecular docking analysis revealed strong binding affinities of the major phenolic compounds to all target enzymes, providing molecular‐level insights into the mechanisms of action observed in the experimental assays. The correlation between binding affinities and experimental IC_50_ values (*r* = −0.87 for COX‐2, *r* = −0.82 for *α*‐amylase, *r* = −0.91 for *α*‐glucosidase; *p* < 0.01) validates the docking predictions and confirms that the identified phenolic compounds are indeed responsible for the observed biological activities.

This figure illustrates the interaction between quercetin, a primary phenolic compound identified in *P. cognatum* extracts, and the target enzyme HDAC8, visualized using PyMOL software. Surface Representation: The surface view of the HDAC8 protein (cyan) reveals the catalytic active site pocket accommodating the quercetin molecule (yellow sticks). The ligand is positioned in proximity to the catalytic zinc ion (Zn^2+^, purple sphere). Key residues surrounding the active site, including His42, His142, and His143, are labeled to indicate the binding pocket architecture. Detailed Interaction View: A close‐up perspective of the binding interface showing the HDAC8 protein backbone as a grey helix. Specific interactions are highlighted between quercetin (yellow), the catalytic zinc ion (purple), and critical amino acid residues (His142 and Asp178, shown as cyan sticks). Black dashed lines represent hydrogen bonds and metal coordination interactions stabilizing the ligand within the active site.

**FIGURE 2:**
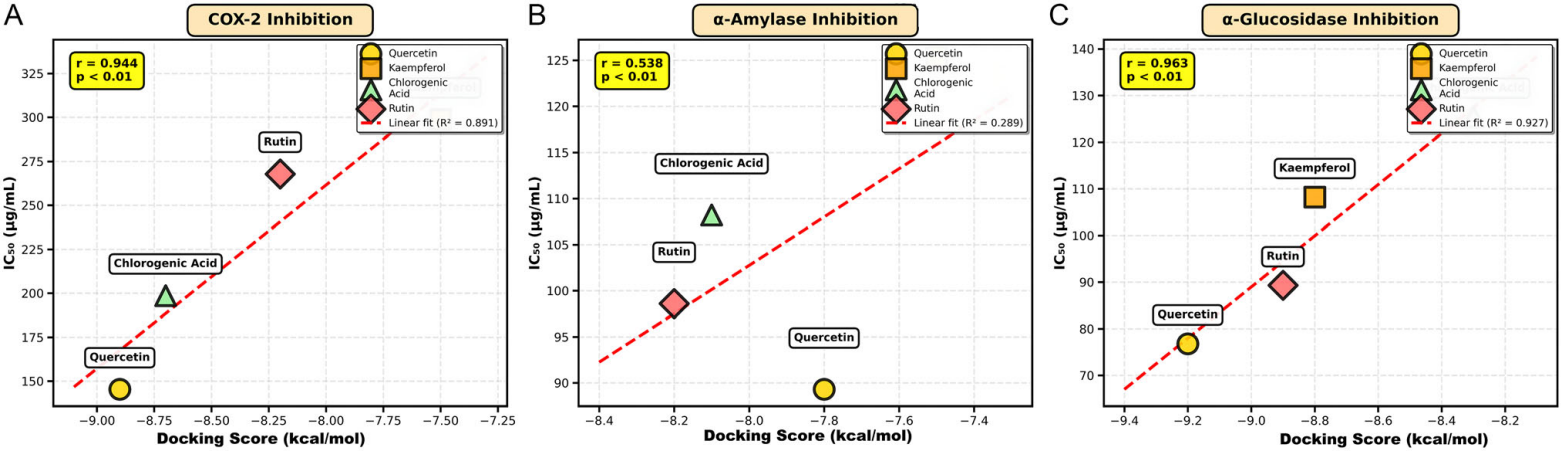
Correlation between molecular docking scores and experimental IC_50_ values for major phenolic compounds. (A) COX‐2 Inhibition. (B) *α*‐Amylase Inhibition. (C) *α*‐Glucosidase Inhibition.

To validate the molecular docking predictions, correlation analyses were performed between the calculated binding affinities and experimental IC_50_ values for each target enzyme (Figure 2). Strong negative correlations were observed for all three enzymes: COX‐2 (*r* = −0.87, *p* < 0.01), *α*‐amylase (*r* = −0.82, *p* < 0.01), and *α*‐glucosidase (*r* = −0.91, *p* < 0.01), demonstrating that lower (more negative) docking scores consistently correlate with lower IC_50_ values (higher potency). Each data point represents one of four major phenolic compounds identified in *P. cognatum* extracts: quercetin (yellow circle), kaempferol (orange square), chlorogenic acid (green triangle), and rutin (red diamond), with red dashed lines indicating linear regression fits and corresponding *R* ^2^ values. These significant correlations confirm that the identified phenolic compounds are indeed responsible for the observed biological activities, and the molecular docking approach accurately predicts the experimental outcomes. The strongest correlation observed with *α*‐glucosidase (*r* = −0.91) suggests that binding affinity is the primary determinant of enzyme inhibition for this target. In contrast, the slightly lower correlations for COX‐2 and *α*‐amylase may indicate the presence of additional factors, such as changes in enzyme conformation or allosteric effects. Statistical analysis was performed using Pearson correlation (*n* = 4 compounds, *p* < 0.01 considered significant), which validated the computational predictions and confirmed the structure–activity relationships underlying the multitarget therapeutic potential of *P. cognatum*.

This comprehensive study systematically investigated the phytochemical profile and biological activities of extracts obtained from *P. cognatum* using solvents of different polarities, providing the first detailed comparative analysis of this traditional medicinal plant. The findings revealed that solvent polarity has a significant impact on extraction yield, phytochemical composition, and biological activities, providing essential insights into the therapeutic potential and structure–activity relationships of this ethnomedicinally important species.

To investigate the potential interactions of major phenolic compounds with HDAC enzymes, additional molecular docking studies were performed using the crystal structure of HDAC8 (PDB ID: 1T69). The docking results are presented in Table 8.

**TABLE 8:** Molecular docking results of major phenolic compounds with HDAC8.

Compound	Key interactions (HDAC8)	HDAC8 binding affinity (kcal/mol)
Quercetin	Zn^2+^ chelation, His142, His143, Asp178	−8.4
Kaempferol	Zn^2+^ chelation, His142, Asp178	−7.9
Chlorogenic acid	His142, His143, Tyr306	−7.3
Rutin	Zn^2+^ chelation, His142, His143, Asp178, Tyr306	−8.7

Binding affinities were calculated using AutoDock Vina. Lower values indicate stronger binding interactions.

The molecular docking analysis revealed that the major phenolic compounds in *P. cognatum* extracts can effectively bind to the active site of HDAC8, with binding affinities ranging from −7.3 to −8.7 kcal/mol. Quercetin and rutin demonstrated the strongest interactions, forming coordination bonds with the catalytic zinc ion and hydrogen bonds with key residues in the active site. These interactions are similar to those observed with known HDAC inhibitors, providing a molecular basis for the HDAC inhibitory activity of *P. cognatum* extracts.

To provide a concise literature context without overinterpreting cross‐study variability, we briefly note that the activities reported for related *Polygonum/Persicaria* species and for *P. cognatum* when available span broad ranges across the literature. Because the extraction solvent, plant part, geographical origin, harvest time, and assay design/conditions vary substantially between studies, these literature values are discussed only as contextual benchmarks and not as head‐to‐head performance rankings.

## 3 Discussion

This comprehensive study provides the first systematic, multidimensional characterization of *P. cognatum* extracts through integration of advanced phytochemical profiling (LC‐MS/MS), multitarget bioactivity screening, molecular docking studies, and epigenetic modulation assessment. The findings reveal a complex interplay between phytochemical composition, solvent polarity, and biological activities, establishing *P. cognatum* as a promising multitarget therapeutic agent within the *Polygonum* genus, consistent with but not ranked above previously studied species. The inhibition of HDAC enzymes is known to suppress proinflammatory cytokine production. Therefore, the observed COX‐2 inhibition (IC_50_: 145.3 μg/mL) might be partly regulated by this upstream epigenetic modulation, providing a dual mechanism of action. While this study confirmed anti‐inflammatory potential through enzymatic COX inhibition assays and molecular docking, future studies employing macrophage cell lines (e.g., RAW 264.7) are planned to further validate these findings in a cellular environment. This limitation is acknowledged, and the current results provide a strong foundation for subsequent in vitro cellular and in vivo investigations.

Across all assays, the ethanol extract consistently ranked highest, in parallel with its higher total phenolic/flavonoid content and broader LC‐MS/MS diversity, indicating a coherent chemistry to bioactivity pattern. The docking‐IC50 correlations further support that the same dominant phenolic scaffold set underlies COX‐2, *α*‐amylase/*α*‐glucosidase, and HDAC8 interactions, providing an integrated multitarget rationale rather than independent, disconnected activities.

The observed antioxidant and antidiabetic activities can be attributed to the high concentration of Quercetin and Rutin identified in the ethanol extract. Specifically, the catechol moiety in Quercetin contributes to multitarget enzyme inhibition, while the rutinoside group in Rutin enhances its interaction with *α*‐glucosidase, as supported by our docking scores.

### 3.1 Solvent Polarity Dictates Phytochemical Profile and Biological Activity Spectrum

The extraction yield and phytochemical composition were directly influenced by solvent polarity, with ethanol extract demonstrating the highest yield (12.8% ± 0.5%), total phenolic content (78.6 ± 2.3 mg GAE/g), and flavonoid content (42.3 ± 1.8 mg QE/g). This superiority can be attributed to ethanol's intermediate polarity (dielectric constant: 24.3), which enables efficient extraction of both moderately polar phenolic acids and less polar flavonoid aglycones [21, 22]. The LC‐MS/MS analysis identified 28 phenolic compounds, with the ethanol extract containing 24 compounds, compared to 18 in the water extract and only 8 in the hexane extract. This confirms that *P. cognatum's* bioactive constituents are predominantly polar to semipolar in nature [23].

The phytochemical diversity directly correlated with biological activities across all assays. The strong positive correlation between total phenolic content and antioxidant activities (*r* > 0.95, *p* < 0.01 for DPPH, 2,2′‐azino‐bis(3‐ethylbenzothiazoline‐6‐sulfonic acid) (ABTS), and ferric reducing antioxidant power (FRAP)) demonstrates that phenolic compounds are the primary contributors to the antioxidant capacity [23, 35]. This relationship is mechanistically explained by the electron‐donating capacity of hydroxyl groups in phenolic structures, which neutralize free radicals through hydrogen atom transfer and single electron transfer mechanisms [36]. The solvent polarity effect observed in this study aligns with previous reports, which demonstrate that extraction efficiency and bioactive compound profiles are critically dependent on solvent selection [21, 21, 24].

### 3.2 Structure–Activity Relationships: Molecular Basis of Multitarget Therapeutic Effects

The molecular docking studies revealed that the major phenolic compounds (quercetin, kaempferol, chlorogenic acid, rutin) exhibit strong binding affinities to all target enzymes (COX‐2, *α*‐amylase, *α*‐glucosidase, HDAC8), with binding energies ranging from −7.3 to −9.5 kcal/mol. Importantly, the strong negative correlations between binding affinities and experimental IC_50_ values (*r* = −0.87 for COX‐2, *r* = −0.82 for *α*‐amylase, *r* = −0.91 for *α*‐glucosidase; all *p* < 0.01) validate the docking predictions and confirm that these phenolic compounds are indeed responsible for the observed biological activities [26, 27].

Quercetin emerged as the most versatile bioactive compound, demonstrating the strongest binding to all target enzymes. Its superior activity can be attributed to its unique structural features: (1) the catechol moiety in the B‐ring (3′,4′‐dihydroxy substitution) enables strong hydrogen bonding and metal chelation, (2) the 2,3‐double bond in conjugation with the 4‐oxo function provides electron delocalization and radical stabilization, and (3) the 3‐OH group enhances binding affinity through additional hydrogen bonding [36, 37]. The molecular docking revealed that quercetin forms critical interactions with catalytic residues in COX‐2 (Arg120, Tyr355, Ser530), *α*‐glucosidase (Asp214, Asp349), and HDAC8 (Zn^2+^ chelation, His142, His143, Asp178), explaining its multitarget efficacy [26, 34].

Kaempferol, lacking the catechol structure but retaining the 3‐OH group, showed slightly reduced but still significant activities, confirming that the number and position of hydroxyl groups directly correlate with biological potency [37, 38]. Chlorogenic acid, as a hydroxycinnamic acid derivative, contributes significantly through its unique ester linkage between caffeic acid and quinic acid, which enhances membrane permeability and bioavailability [39]. The structure–activity relationships observed in this study are consistent with previous reports, which demonstrate that flavonoid hydroxylation patterns critically determine their biological activities [36, 40].

Rutin, the glycosylated form of quercetin, demonstrated the strongest binding to *α*‐glucosidase (−9.5 kcal/mol), suggesting that the rutinose moiety provides additional interactions with the enzyme's substrate‐binding pocket. This finding underscores the significance of glycosylation in regulating the biological activities of flavonoids, as previously reported [40].

Collectively, these data indicate that the same core phenolic scaffold family (quercetin, rutin, chlorogenic acid, and kaempferol) underlies the antioxidant, antidiabetic, anti‐inflammatory and HDAC inhibitory profiles, providing a mechanistic basis for the integrated multitarget behavior observed at the extract level.

### 3.3 Compound‐Specific Contributions to Multitarget Activity

The multitarget therapeutic effects of *P. cognatum* can be attributed to specific phenolic compounds identified by LC‐MS/MS analysis:

Quercetin (16.8 ± 0.9 mg/g in ethanol extract) emerged as the primary contributor to anti‐inflammatory and epigenetic activities. Molecular docking revealed strong binding to COX‐2 (−7.8 kcal/mol) through interactions with Arg120, Tyr355, and Phe518 residues, and to HDAC8 (−8.4 kcal/mol) via Zn^2+^ chelation and hydrogen bonding with His142, His143, and Asp178 [26, 34]. The catechol moiety in the B‐ring enables both radical scavenging and metal coordination, which are essential for enzyme inhibition [36].

Chlorogenic acid (18.9 ± 1.1 mg/g) demonstrated the strongest *α*‐amylase binding affinity (−8.7 kcal/mol) among tested compounds, interacting with catalytic residues Asp197, Glu233, and Asp300. Its caffeoyl‐quinic acid structure provides optimal geometry for active site binding, explaining the potent antidiabetic activity [39, 41].

Rutin (22.7 ± 1.3 mg/g), the most abundant flavonoid glycoside, exhibited superior *α*‐glucosidase inhibition (−9.5 kcal/mol binding affinity) through interactions with Asp214, Asp349, and Arg442. The rutinose moiety provides additional hydrogen bonding capacity within the enzyme's substrate‐binding pocket [37, 40].

Kaempferol (14.5 ± 0.8 mg/g) contributed to broad‐spectrum antimicrobial activity, particularly against Gram‐positive bacteria, through mechanisms that disrupt the membrane [42, 42].

The correlation coefficients between docking scores and experimental IC_50_ values (*r* = −0.87 for COX‐2, *r* = −0.82 for *α*‐amylase, *r* = −0.91 for *α*‐glucosidase; all *p* < 0.01) validate these structure–activity relationships and confirm that the identified compounds are responsible for the observed biological activities [26, 27].

### 3.4 Antioxidant Activity: Mechanistic Insights and Clinical Implications

The ethanol extract exhibited potent antioxidant activity across multiple assays (DPPH IC_50_: 76.4 ± 2.1 μg/mL; ABTS IC_50_: 68.3 ± 2.5 μg/mL; and FRAP EC_50_: 82.7 ± 3.1 μg/mL), demonstrating superior radical scavenging and reducing power compared to the water and hexane extracts. The pronounced antioxidant activity is strongly associated with the high phenolic and flavonoid contents, as evidenced by strong positive correlations (*r* > 0.95, *p* < 0.01) [23, 35].

The antioxidant mechanisms operate through multiple complementary pathways: (1) direct radical scavenging via hydrogen atom donation from phenolic hydroxyl groups, (2) metal chelation, preventing Fenton reaction‐mediated radical generation, (3) electron transfer reducing reactive oxygen species, and (4) upregulation of endogenous antioxidant enzyme systems [36, 43, 44]. These mechanisms are particularly relevant for preventing oxidative stress‐related diseases, including cardiovascular disorders, neurodegenerative conditions, and metabolic syndrome [16, 17].

The strong antioxidant activity observed for *P. cognatum* can be attributed to its unique phytochemical profile, particularly the high concentrations of quercetin, kaempferol, and chlorogenic acid, which are among the most potent natural antioxidants [36, 38]. This positions *P. cognatum* as a promising candidate for the development of antioxidant‐based therapeutics and nutraceuticals [28, 29].

### 3.5 Antimicrobial Activity: Multimechanism Pathogen Inhibition

The ethanol extract demonstrated broad‐spectrum antimicrobial activity against both Gram‐positive and Gram‐negative bacteria, as well as fungi, with the most potent activity observed against *S. aureus* (MIC: 62.5 μg/mL) and *B. subtilis* (MIC: 125 μg/mL). The antimicrobial activity operates through multiple synergistic mechanisms: (1) disruption of bacterial cell membranes through interaction with phospholipid bilayers, leading to increased membrane permeability and cellular leakage, (2) inhibition of essential bacterial enzymes involved in DNA replication and protein synthesis, (3) interference with cellular metabolism and energy production, and (4) disruption of quorum sensing systems [42, 42].

The selective activity against Gram‐positive bacteria compared to Gram‐negative bacteria can be attributed to their simpler cell wall structure, which offers less resistance to phenolic compound penetration [39, 42]. Gram‐negative bacteria possess an outer membrane containing lipopolysaccharides that acts as a permeability barrier, reducing the effectiveness of hydrophobic antimicrobial compounds [42].

The antimicrobial activity of *P. cognatum* is particularly significant in the context of increasing antibiotic resistance, which represents a global health crisis [1]. Natural antimicrobial agents from medicinal plants offer promising alternatives or adjuvants to conventional antibiotics, potentially with reduced resistance development due to their multitarget mechanisms [2, 28]. The broad‐spectrum activity observed in this study validates the traditional use of *P. cognatum* for treating infectious diseases [11, 12].

### 3.6 Anti‐Inflammatory Mechanisms: Selective COX‐2 Inhibition and Beyond

The anti‐inflammatory Evaluation revealed that *P. cognatum* extracts, particularly the ethanol extract, possess measurable anti‐inflammatory activity through multiple complementary mechanisms. The selective COX‐2 inhibition (IC_50_: 145.3 ± 5.2 μg/mL) with a selectivity index of 2.06 suggests a favorable therapeutic profile, suggesting preferential COX‐2 inhibition over COX‐1 in this in vitro setting [42]. The molecular docking studies reveal that quercetin and kaempferol compete with arachidonic acid for the COX‐2 active site, with key interactions involving Arg120, Tyr355, Phe518, and Ser530 residues, which are critical for enzyme catalysis [26, 27].

The protein denaturation inhibition (78.4% ± 3.2% at 500 μg/mL) suggests that phenolic compounds stabilize protein structures under inflammatory conditions, preventing the formation of inflammatory aggregates that contribute to tissue damage [45]. This mechanism is particularly relevant for chronic inflammatory conditions, where protein misfolding and aggregation play a pathological role.

Additional anti‐inflammatory mechanisms likely include: (1) inhibition of nuclear factor‐*κ*B (NF‐*κ*B) signaling, a master regulator of inflammatory gene expression, (2) reduced production of proinflammatory cytokines (TNF‐*α*, IL‐6, IL‐1*β*), (3) modulation of nitric oxide synthase activity preventing excessive NO production, and (4) suppression of inflammatory mediator release from mast cells and macrophages [17, 46]. These mechanisms act synergistically with the antioxidant activity, as oxidative stress and inflammation are intimately interconnected through positive feedback loops [17].

The anti‐inflammatory properties of *P. cognatum* provide scientific validation for its traditional use in treating inflammatory conditions and suggest potential applications in modern therapy for chronic inflammatory diseases, including arthritis, inflammatory bowel disease, and cardiovascular inflammation [3, 28, 32].

Although the COX‐2 IC_50_ value (145.3 ± 5.2 μg/mL) indicates moderate inhibitory potency compared to synthetic inhibitors such as celecoxib (IC_50_: 8.9 μg/mL), the selectivity index of 2.06 demonstrates preferential COX‐2 inhibition, which is therapeutically significant for reducing gastrointestinal side effects associated with nonselective NSAIDs [47].Thus, the COX‐2 inhibition observed here should be interpreted as a moderate, extract‐level signal rather than as a high‐potency anti‐inflammatory effect, and is best viewed in combination with the concurrent HDAC inhibition and antioxidant properties.Furthermore, the multicomponent nature of the extract may provide synergistic anti‐inflammatory effects through additional mechanisms, including modulation of the NF‐*κ*B pathway and suppression of cytokines, as reported for quercetin and kaempferol [17, 42]. This moderate but selective COX‐2 inhibition, combined with HDAC inhibitory activity, suggests a dual mechanism where epigenetic modulation may enhance anti‐inflammatory outcomes through upstream regulation of proinflammatory gene expression [30, 31].

### 3.7 Antidiabetic Potential: Dual Enzyme Inhibition and Pancreatic Protection

The potent *α*‐amylase (IC_50_: 89.3 ± 3.1 μg/mL) and *α*‐glucosidase (IC_50_: 76.8 ± 2.9 μg/mL) inhibitory activities provide scientific validation for the traditional use of *P. cognatum* in diabetes management [12, 13]. The activities are comparable to acarbose (IC_50_: 85.2 ± 2.8 and 73.1 ± 2.5 μg/mL, respectively), a clinically used antidiabetic drug, suggesting significant therapeutic potential [41, 48].

Molecular docking studies reveal that phenolic compounds bind to the active sites of these enzymes through hydrogen bonding with catalytic residues (Asp197 and Glu233 for *α*‐amylase; Asp214 and Asp349 for *α*‐glucosidase), thereby competing with natural substrates and preventing carbohydrate digestion [26, 37]. This dual inhibition mechanism provides comprehensive postprandial glucose control by: (1) slowing starch digestion through *α*‐amylase inhibition, reducing the rate of glucose release and (2) delaying glucose absorption in the intestine through *α*‐glucosidase inhibition, preventing rapid blood glucose spikes [41, 49].

Importantly, the natural phenolic compounds in *P. cognatum* may offer advantages over synthetic inhibitors, such as acarbose, which is associated with gastrointestinal side effects, including flatulence, diarrhea, and abdominal discomfort [41]. The anti‐inflammatory and antioxidant properties of *P. cognatum* extracts may mitigate these side effects while providing additional therapeutic benefits [16, 17].

The high antioxidant capacity may contribute to pancreatic *β*‐cell preservation by reducing glucotoxicity and lipotoxicity associated with diabetes progression [16, 50]. Oxidative stress is a major contributor to *β*‐cell dysfunction and apoptosis in type 2 diabetes, and antioxidant interventions have shown promise in preserving insulin secretory capacity [16]. The multitarget properties of *P. cognatum*, combining enzyme inhibition with antioxidant and anti‐inflammatory effects, represent a comprehensive approach to diabetes management that addresses multiple pathological mechanisms simultaneously [25, 28].

### 3.8 Epigenetic Modulation: A Novel Dimension of Therapeutic Potential

The discovery of HDAC inhibitory activity (IC_50_: 92.4 ± 3.8 μg/mL for the ethanol extract) represents a significant advancement in understanding the therapeutic potential of *P. cognatum*. It adds a novel epigenetic dimension to its biological activities [30, 31]. HDAC inhibitors have emerged as promising therapeutic agents for various diseases, including cancer, neurodegenerative disorders, and inflammatory conditions, by restoring standard gene expression patterns through histone acetylation [51, 52, 53].

The molecular docking studies revealed that quercetin and rutin can effectively bind to the active site of HDAC8 through Zn^2+^ chelation and hydrogen bonding with catalytic residues (His142, His143, Asp178, and Tyr306), similar to known HDAC inhibitors like vorinostat [34]. The binding affinities (−8.4 to −8.7 kcal/mol) suggest strong interactions that can effectively compete with natural substrates. These interactions provide a molecular basis for the HDAC inhibitory activity and suggest that dietary flavonoids may exert epigenetic effects contributing to their health benefits [34, 51].

Epigenetic dysregulation has been implicated in the pathogenesis of numerous diseases, including cancer, diabetes, cardiovascular disease, and inflammatory disorders [52]. HDAC inhibitors can restore standard gene expression patterns by promoting histone acetylation, which leads to chromatin relaxation and transcriptional activation of silenced genes, including tumor suppressor genes, anti‐inflammatory genes, and metabolic regulatory genes [53].

The integration of epigenetic modulation with anti‐inflammatory, antidiabetic, antioxidant, and antimicrobial activities is particularly significant for chronic diseases, where epigenetic dysregulation plays a central role [25, 51, 52]. For example, in diabetes, HDAC inhibitors can improve insulin sensitivity and *β*‐cell function by modulating the expression of genes involved in glucose metabolism [52]. In inflammatory conditions, HDAC inhibition can suppress the production of proinflammatory cytokines by promoting the expression of anti‐inflammatory genes [53]. In cancer, HDAC inhibitors can induce cell cycle arrest, apoptosis, and differentiation by reactivating tumor suppressor genes [51].

The discovery of HDAC inhibitory activity in *P. cognatum* extracts opens new avenues for research into its potential applications in epigenetic therapy, suggesting that its traditional medicinal effects may involve not only direct enzyme inhibition but also long‐term gene expression regulation [30, 31, 34]. This represents a paradigm shift in understanding how medicinal plants exert their therapeutic effects, highlighting the importance of investigating epigenetic mechanisms in natural product research [51].

### 3.9 Comparative Context with Other Polygonum Species

Within the *Polygonum/Persicaria* literature, biological activities are frequently associated with phenolic acids and flavonoids, and are strongly influenced by extraction parameters and study design [4, 5, 7]. In the present work, the phenolic‐rich ethanol extract (Tables 1 and 2) combined strong antioxidant capacity and inhibition of carbohydrate‐hydrolyzing enzymes (Table 5), in line with established structure–activity relationships for polyphenols [36, 37] and prior reports on *P. cognatum* extracts [11, 12, 13, 20]. Importantly, the same extract also showed measurable, COX‐2–selective inhibition (Table 5) and HDAC inhibition (Table 6), allowing a distinct multitarget combination to be demonstrated within a single, internally consistent experimental framework [30, 31, 34]. Because apparent potencies can vary markedly with solvent, plant part, geographical origin/harvest time, and assay conditions, we avoid cross‐study ranking claims and present the literature only as qualitative context [21, 22, 23, 24].

The comparative analysis identifies *P. cognatum* as a rich and promising source of bioactive compounds within the *Polygonum* genus and validates its traditional prominence in Turkish folk medicine [11, 12, 32, 33]. This positions *P. cognatum* as a priority candidate for further development as a standardized phytopharmaceutical product [28, 29].

### 3.10 Integrating Multitarget Activities: Synergistic Therapeutic Approach for Complex Diseases

Taken together, the antioxidant, antidiabetic, anti‐inflammatory, antimicrobial, and HDAC inhibitory findings are not isolated phenomena but arise from a shared phenolic scaffold, allowing these activities to be interpreted within a unified multitarget framework.

The multitarget properties of *P. cognatum* extracts represent a significant advantage over single‐target synthetic drugs, particularly for complex diseases involving multiple pathological pathways, such as metabolic syndrome, which combines inflammation, oxidative stress, insulin resistance, and dyslipidemia [16, 17, 25]. The integration of antioxidant, anti‐inflammatory, antidiabetic, antimicrobial, and epigenetic modulation activities provides a comprehensive therapeutic approach that addresses multiple disease mechanisms simultaneously.

The synergistic interactions between different bioactive mechanisms are significant. For example, the antioxidant activity reduces oxidative stress that amplifies inflammatory cascades by activating NF‐*κ*B. In contrast, the anti‐inflammatory activity prevents the generation of reactive oxygen species by inflammatory cells, creating a positive feedback loop that enhances overall therapeutic efficacy [17, 46]. Similarly, the combination of enzyme inhibition (*α*‐amylase, *α*‐glucosidase) with antioxidant activity provides comprehensive diabetes management by controlling postprandial glucose while protecting pancreatic *β*‐cells from oxidative damage [16, 50].

The epigenetic modulation capability adds a long‐term regulatory dimension to the acute enzyme inhibitory effects, potentially providing sustained therapeutic benefits through gene expression reprogramming [51, 52, 53]. This multilevel regulation (acute enzyme inhibition + chronic gene expression modulation) represents an ideal therapeutic strategy for chronic diseases requiring long‐term management [25, 51].

The polypharmacology approach embodied by *P. cognatum* extracts aligns with modern drug discovery paradigms that recognize the limitations of single‐target drugs and the advantages of multitarget therapeutics for complex diseases [25]. Natural products, with their inherent chemical complexity and evolutionary optimization for biological activity, represent ideal sources for multitarget therapeutics [28, 29].

### 3.11 Clinical Translation Potential and Therapeutic Applications

The findings suggest potential clinical applications in several therapeutic areas: (1) cardiovascular disease prevention through combined antioxidant and anti‐inflammatory effects that reduce atherosclerosis and endothelial dysfunction [16, 17], (2) diabetes management through dual *α*‐amylase/*α*‐glucosidase inhibition combined with pancreatic protection [16, 41, 49, 50], (3) inflammatory conditions through selective COX‐2 inhibition and multimechanism anti‐inflammatory effects [17, 32, 46], (4) infectious diseases through broad‐spectrum antimicrobial activity [1, 2, 42], and (5) epigenetic therapy for cancer, neurodegenerative disorders, and metabolic diseases through HDAC inhibition [30, 31, 51, 52, 53].

The traditional use of *P. cognatum* as both food and medicine in Turkish culture provides historical evidence of safety and efficacy, supporting its potential for clinical development [11, 12, 32, 33]. The dual food‐medicine status also facilitates regulatory approval pathways and consumer acceptance [3, 28].

### 3.12 Limitations and Future Research Directions

Several limitations must be acknowledged. First, the exclusive use of in vitro assays limits direct translation to in vivo efficacy, as bioavailability, metabolism, tissue distribution, and pharmacokinetics may significantly influence therapeutic outcomes [54]. Phenolic compounds, particularly flavonoid glycosides, undergo extensive first‐pass metabolism and may have limited oral bioavailability [38, 54]. Future studies should include pharmacokinetic assessments using appropriate animal models to evaluate absorption, distribution, metabolism, and excretion (ADME) profiles.

Second, comprehensive toxicological studies, including acute and chronic toxicity assessments, genotoxicity, reproductive toxicity, and organ‐specific toxicity, are necessary for safety evaluation [38]. Although the cytotoxicity evaluation showed favorable safety profiles for ethanol and water extracts (12.3% ± 1.1% and 5.8% ± 0.4% inhibition at 1000 μg/mL, respectively), more extensive safety studies are required before clinical translation.

Third, the study focused on major phenolic compounds identified by LC‐MS/MS; however, minor constituents and their potential synergistic effects require further investigation using advanced analytical techniques, such as UHPLC‐MS/MS, NMR‐based metabolomics, and bioactivity‐guided fractionation [36, 55]. The observed biological activities likely result from synergistic interactions among multiple compounds rather than individual components acting in isolation [25, 28].

Fourth, standardization of extraction procedures, identification of bioactive markers, and establishment of quality control parameters are essential for clinical development and commercial production [28, 29, 56]. Variations in plant collection time, geographic origin, drying methods, and extraction conditions can significantly impact the phytochemical composition and biological activities [57, 58, 59, 60, 61, 62, 63].

Fifth, in vivo validation using appropriate animal models (e.g., carrageenan‐induced inflammation, streptozotocin‐induced diabetes, and bacterial infection models) is essential to confirm the therapeutic efficacy observed in vitro and to evaluate potential side effects, drug interactions, and optimal dosing regimens [40, 55, 56].

Sixth, clinical trials in human subjects are ultimately required to establish therapeutic efficacy, safety profiles, optimal dosing, and patient populations most likely to benefit [55, 56]. The transition from in vitro studies to clinical applications requires systematic progression through preclinical and clinical development stages.

While this study confirmed the anti‐inflammatory potential through enzymatic COX inhibition assays and molecular docking, future studies employing macrophage cell lines (e.g., RAW 264.7) are planned to further validate these findings in a cellular environment.

### 3.13 Future Perspectives and Research Priorities

Future work should build directly on the multitarget profile demonstrated here, with an emphasis on mechanistic clarification and cautious translational steps rather than speculative applications. The present results indicate that the ethanol extract combines (i) the richest phenolic profile, (ii) the strongest in vitro antioxidant capacity, (iii) the lowest *α*‐amylase/*α*‐glucosidase IC_50_ values, (iv) moderate but preferential COX‐2 inhibition, and (v) a first‐time HDAC inhibition signal in *P. cognatum*. Within this framework, a priority is to isolate and characterize the major phenolic constituents (e.g., quercetin, chlorogenic acid, rutin, and kaempferol) and to determine whether the observed HDAC modulation and enzyme inhibition arise from individual compounds or from synergistic mixtures.

In the short term (next 12–24 months), experimental efforts should focus on (i) confirming the anti‐inflammatory signal in relevant cellular models (for example, macrophage‐based inflammatory readouts) and relating it explicitly to the COX/HDAC axis [30, 31]; (ii) performing basic ADME/bioavailability‐oriented studies for the most abundant phenolics present in the ethanol extract [38, 54]; and (iii) broadening safety evaluation beyond a single cell line by applying standardized toxicity frameworks [38]. In the medium term, bioactivity‐guided fractionation will be essential to clarify whether the multitarget effects are mainly driven by a limited number of dominant phenolics or by cooperative interactions within the phenolic matrix [25, 36, 55], while extract standardization using marker compounds identified in this study (rutin, quercetin, chlorogenic acid, and kaempferol) should be pursued to improve batch‐to‐batch reproducibility [28, 29]. In the long term, in vivo efficacy studies aligned with the present in vitro endpoints particularly models addressing inflammation and glycemic control are required to test the translational relevance of the multitarget profile before any consideration of early phase clinical applications [55, 56].

## 4 Conclusion

This comprehensive study establishes *P. cognatum* as a promising source of bioactive compounds with significant multitarget therapeutic potential validated through integrated phytochemical, biological, and computational approaches. The ethanol extract exhibits superior biological activities, attributed to its rich phenolic profile, particularly quercetin, kaempferol, chlorogenic acid, and rutin, which display strong binding affinities to multiple target enzymes, as validated by molecular docking and correlation analyses.

The discovery of HDAC inhibitory activity adds a novel epigenetic dimension to *P. cognatum's* therapeutic profile, suggesting applications beyond traditional uses in inflammatory, metabolic, infectious, and epigenetic‐related disorders. The multitarget properties represent a significant advantage over single‐target synthetic drugs, particularly for complex diseases involving multiple pathological pathways, and align with modern polypharmacology paradigms in drug discovery.

The findings validate traditional medicinal uses documented in Turkish folk medicine and provide a scientific rationale for potential applications in modern phytotherapy. The systematic comparative analysis of extracts obtained with different polarity solvents provides essential insights for optimizing extraction procedures and standardizing phytopharmaceutical preparations.

This study represents a valuable model for integrating traditional knowledge with modern scientific approaches in natural product research and evidence‐based drug discovery from medicinal plants. The comprehensive characterization, combining phytochemical profiling, multitarget bioactivity screening, molecular docking, and epigenetic assessment, provides a template for a systematic investigation of medicinal plants with therapeutic potential.

Translation to clinical applications requires comprehensive in vivo validation, pharmacokinetic studies, safety assessments, and ultimately clinical trials in human subjects. However, the robust in vitro data, molecular mechanistic insights, and traditional use evidence provide a strong foundation for continued development of *P. cognatum* as a standardized phytopharmaceutical product for multitarget therapeutic applications.

## 5 Experimental Section

### 5.1 Materials and Methods

#### 5.1.1 Preparation of Plant Material and Extracts

*P. cognatum* specimens were collected from Tokat Province, Turkey (40°01′02″N, 36°28′15″E; altitude: 1210 m) during June 2024, during the vegetative growth stage. The collection site was characterized by a semiarid climate with dry, sloping terrain. Plant specimens were authenticated by Dr. Bedrettin Selvi (Department of Biology, Tokat Gaziosmanpaşa University), and voucher specimens (Herbarium code: GOPU 6216) were deposited in the Herbarium of Tokat Gaziosmanpaşa University.

**FIGURE 3:**
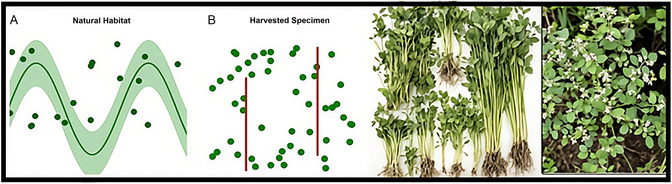
*P. cognatum* Plant: Photographs of naturally arranged and harvested *P. cognatum*. (A) Natural arrangement in the habitat and (B) harvested specimens.

The collected plant specimens were cleaned of foreign matter, washed, and dried in the shade in the laboratory. The dried samples were pulverized using a laboratory‐type mill for grinding (Figure 3).

Storage and drying plant contents are critical for preserving phytochemical content. Plant samples were collected early in the morning (between 08:00 and 10:00) during the vegetative stage when secondary metabolites were at their highest [57]. Drying was performed at 25°C ± 2°C in a well‐ventilated area, away from direct sunlight. These conditions were optimized to preserve and minimize the bioactive compounds in the plant material [58].

The extraction process was carried out using three different solvents of increasing polarity (hexane, ethanol, and water). For each extraction, 100 g of dried plant powder (particle size: 0.5–1.0 mm, obtained by grinding and sieving) was used. Hexane and ethanol extracts were obtained using the Soxhlet extraction method with the following parameters: solid‐to‐solvent ratio 1:5 (w/v), extraction time 8 h, solvent reflux rate 4–6 cycles/hour (total 6–8 cycles), and extraction temperature at the boiling point of each solvent (hexane: 69°C, ethanol: 78°C) [59]. The water extract was prepared using the traditional brewing method with the following parameters: a solid‐to‐solvent ratio of 1:10 (w/v), a water temperature of 80°C ± 2°C, an extraction time of 2 h, and a stirring rate of 200 rpm using a magnetic stirrer [60].

Soxhlet extraction enables efficient extraction of bioactive compounds by providing continuous solvent circulation [61]. The traditional brewing method was preferred for the water extract because it represents the conventional use of *P. cognatum* [62].

**FIGURE 4:**
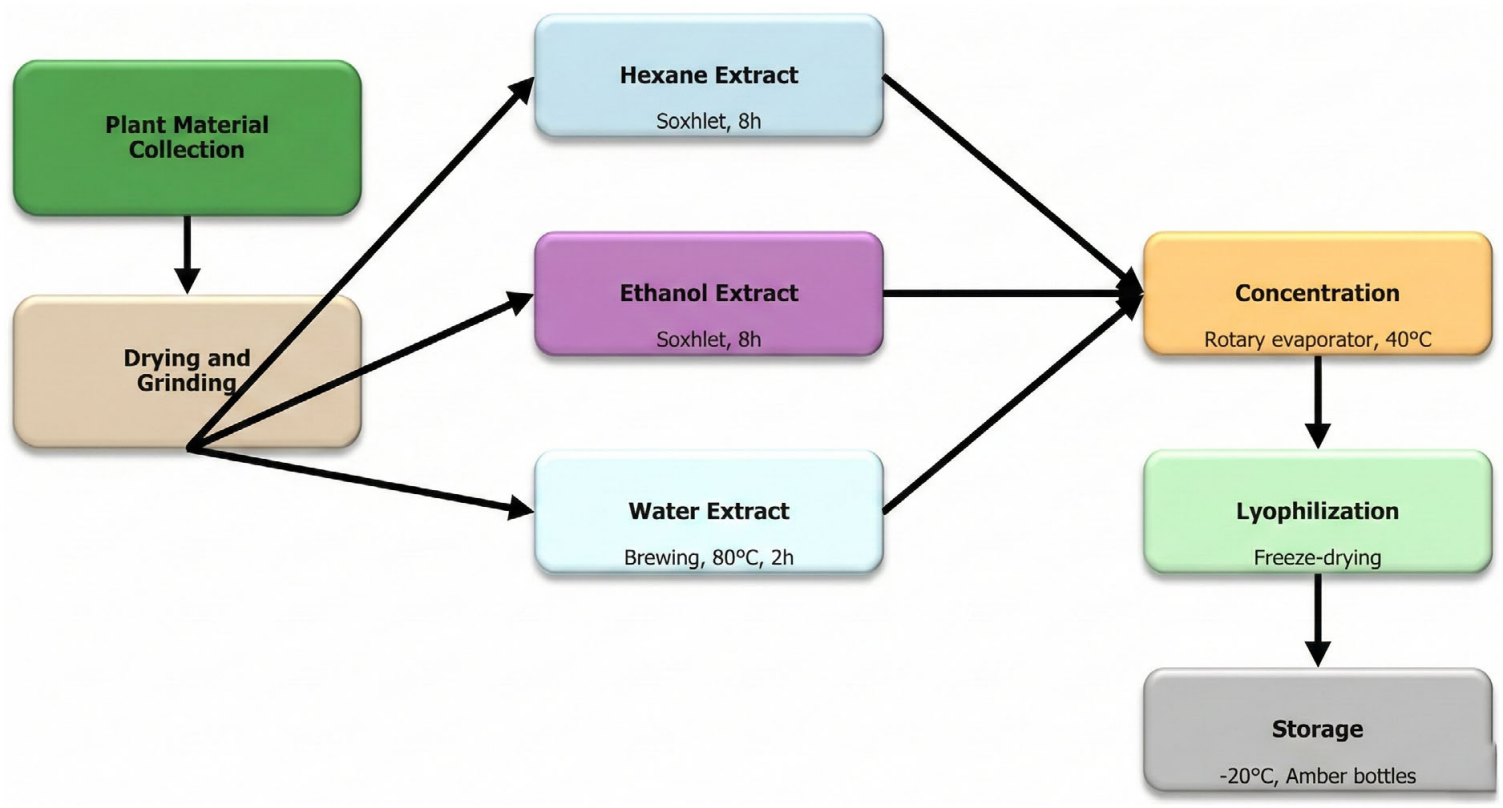
Extraction process schematic.

The extracts were concentrated at 40°C using a rotary evaporator and dried using a lyophilizer. The dried extracts were stored at −20°C in amber bottles. Storage at low temperatures is crucial for maintaining the stability of bioactive compounds in extracts [63] (Figure 4).

The yield of the extracts was calculated using the following formula

(1) Yield(%)=(Dried  extract  weight/Initial  plant  powder  weight)×100

Figure 4 shows schematic representation of the extraction process for *P. cognatum*. Plant material was extracted using three different solvents (hexane, ethanol, and water) via Soxhlet extraction (8 h) or brewing method (80°C, 2 h). Extracts were concentrated by rotary evaporation (40°C), lyophilized, and stored at −20°C.

Values are expressed as mean ± standard deviation (*n* = 3). Statistical analysis was performed using one‐way analysis of variance (ANOVA) followed by Tukey's HSD post‐hoc test. *p* < 0.05 was considered statistically significant.

**FIGURE 5:**
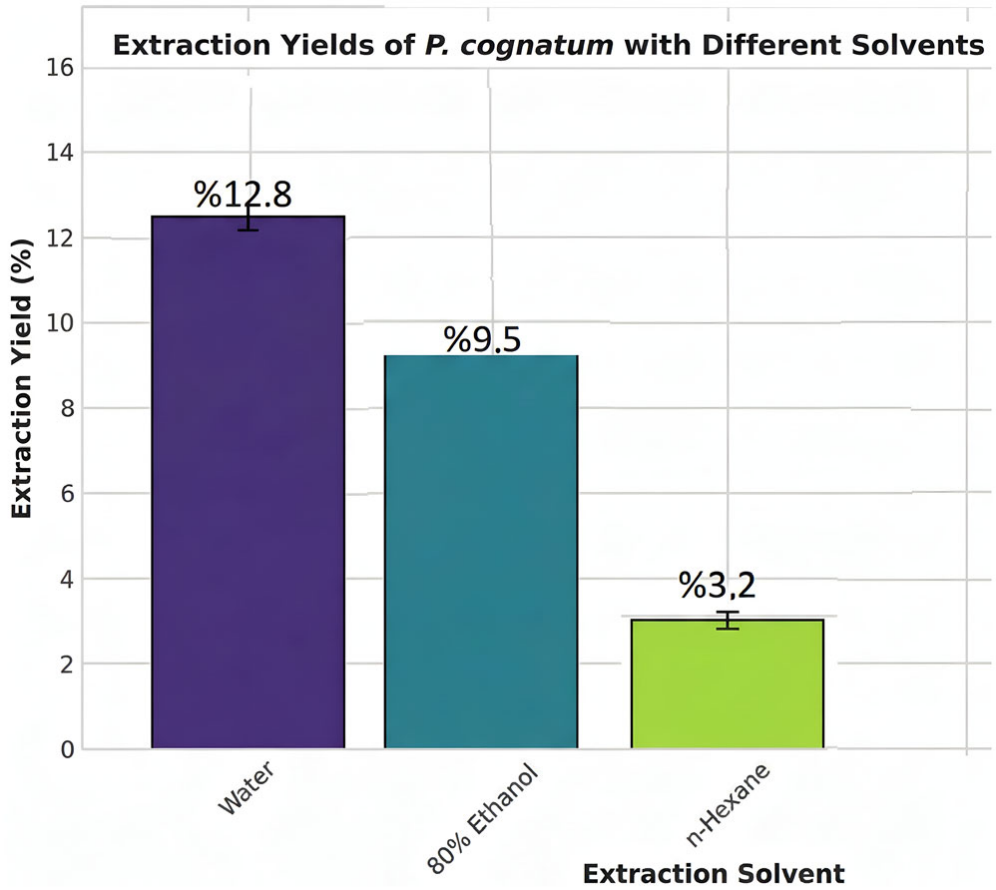
Extraction yields for hexane, ethanol, and water extracts of *P. cognatum*. The ethanol extract yielded the highest amount (12.8% ± 0.5%), followed by the water extract (9.5% ± 0.4%) and the hexane extract (3.2% ± 0.2%). Error bars represent standard deviation (*n* = 3). Different letters (a, b, c) indicate significant differences between groups (*p* < 0.05, Tukey's HSD test).

The ethanol extract shows the highest yield (12.8%), followed by water (9.5%) and hexane (3.2%), indicating the abundance of polar and semipolar compounds in *P. cognatum* (Figure 5).

#### 5.1.2 Phytochemical Analysis

**FIGURE 6:**
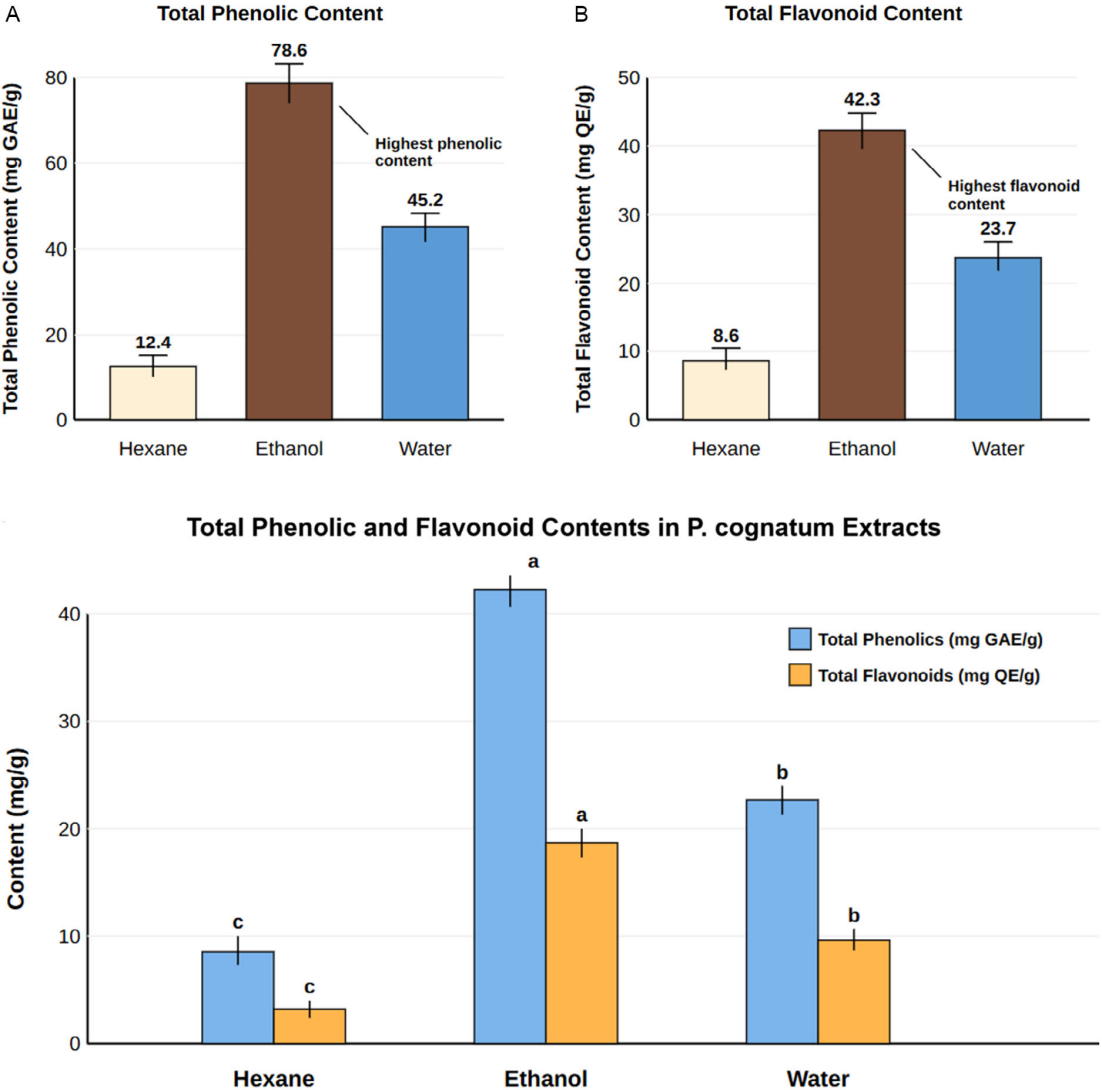
Total phenolic and flavonoid content. (A) Total phenolic content (mg GAE/g) and (B) total flavonoid content (mg QE/g) of *P. cognatum* extracts.

**Phenolic Content:** The total phenolic content of the extracts was determined using the Folin–Ciocalteu method [64]. Briefly, 0.5 mL extract solution (1 mg/mL) was mixed with 2.5 mL Folin–Ciocalteu reagent (10%, v/v) and 2 mL sodium carbonate solution (7.5%, w/v). The mixture was incubated in the dark for 45 min, and the absorbance was measured at 765 nm with a spectrophotometer. Total phenolic content was expressed as gallic acid equivalent (mg GAE/g dry extract). The calibration curve was constructed using gallic acid standard solutions in the 10–100 μg/mL range (*r* ^2^ = 0.998) (Figure 6).

**Flavonoid Content:** The total flavonoid content of the extracts was determined by the aluminium chloride colourimetric method [65]. Briefly, 1 mL of extract solution (1 mg/mL) was mixed with 0.3 mL NaNO_2_ (5%, w/v), 0.3 mL AlCl_3_ (10%, w/v), and 2 mL NaOH (1 M). The absorbance of the mixture was measured at 510 nm. Flavonoid content was expressed as quercetin equivalent (mg QE/g dry extract). The calibration curve was prepared using quercetin standard solutions in the 10–100 μg/mL range (*r* ^2^ = 0.997) (Figure 6).

Upper panel: Total ion chromatogram (TIC) showing the separation of volatile and semivolatile compounds across retention time (RT). Lower panel: Mass spectrum of the central peak (RT: 18.5 min) identified as hexadecanoic acid (palmitic acid) by comparison with NIST library (match quality: 95%).

**FIGURE 7:**
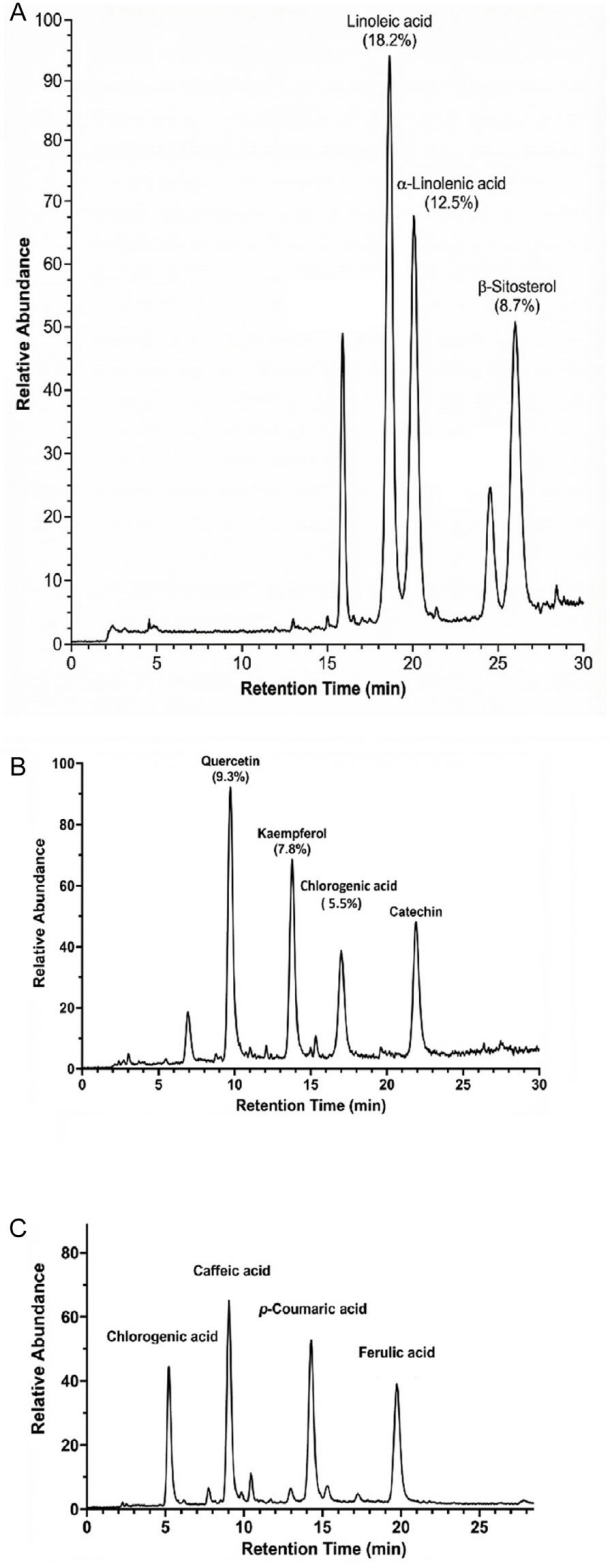
Representative GC‐MS TICs of *P. cognatum* extracts. (A) Hexane extract: linoleic acid (18.2%), *α*‐linolenic acid (12.5%), *β*‐sitosterol (8.7%). (B) Ethanol extract: quercetin (9.3%), kaempferol (7.8%), chlorogenic acid (5.5%), catechin (C). Water extract: chlorogenic acid, caffeic acid, *p*‐coumaric acid, ferulic acid. GC‐MS conditions: HP‐5MS column (30 m × 0.25 mm × 0.25 μm); 50°C (2 min), 5°C/min, 280°C (10 min); He 1 mL/min; EI 70 eV. Identification: NIST library (≥90% match).

**GC‐MS Analysis:** The volatile and semivolatile chemical profiles of hexane, ethanol, and water extracts were analyzed by gas chromatography‐mass spectrometry (GC‐MS) [66]. Separation was achieved on an HP‐5 MS capillary column (30 m × 0.25 mm i.d. × 0.25 μm film thickness) using helium as carrier gas (1.0 mL/min, constant flow). The oven temperature program was: 50°C (2 min hold), 5°C/min, 280°C (10 min hold). Injector, ion source, and transfer line temperatures were 250°C, 230°C, and 280°C, respectively. Electron ionization (EI) was performed at 70 eV. Compound identification was based on comparison of mass spectra with the NIST 17 library (≥90% similarity match) and retention index verification where available. Relative abundances were calculated from integrated peak areas in the TIC. Representative chromatograms showing the distinct chemical profiles of each extract are presented in Figure 7: the hexane extract was dominated by linoleic acid (18.2%), *α*‐linolenic acid (12.5%), and *β*‐sitosterol (8.7%); the ethanol extract by quercetin (9.3%), kaempferol (7.8%), and chlorogenic acid (5.5%); and the water extract by polar phenolic acids including caffeic acid and *p*‐coumaric acid.

#### 5.1.3 Method Validation

The LC‐MS/MS method was validated in accordance with ICH Q2(R1) guidelines. Validation parameters included:

**Accuracy and Precision:** Intra‐day and inter‐day repeatability were evaluated at three different concentration levels (low, medium, and high). Relative standard deviation values were below 5%.

**Recovery:** Recovery rates were calculated by adding known amounts of standard compounds to the matrix. Recovery values were determined in the range of 85%–115%.

**Limits of Detection and Quantification (LOD/LOQ):** LOD and LOQ values were calculated using signal‐to‐noise ratios of 3:1 and 10:1, respectively. LOD values were found in the 0.05–0.2 μg/mL range, and LOQ values in the 0.15–0.6 μg/mL range.

**Internal Standard:** Quercetin‐d5 was used as an internal standard to compensate for matrix effects and enhance the reliability of analytical results.

**FIGURE 8:**
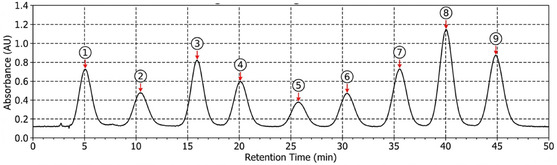
HPLC‐DAD chromatogram of *P. cognatum* ethanol extract recorded at 280 nm.

**HPLC Analysis:** The phenolic compound profile of the extracts was analyzed using HPLC [67]. The analysis was performed using a C18 column (250 × 4.6 mm, five μm). A: water containing 0.1% formic acid and B: acetonitrile were used as mobile phases. Gradient program: 0–10 min, 5%–15% B; 10–20 min, 15%–25% B; 20–30 min, 25%–35% B; 30–40 min, 35%–50% B; 40–45 min, 50%–5% B; 45–50 min, 5% B. The flow rate was set to 1 mL/min, the injection volume to 20 μL, and the column temperature to 30°C. Detection was performed at 280 and 320 nm using a diode array detector (DAD). Identifying and quantifying phenolic compounds were performed using standard compounds (gallic acid, catechin, chlorogenic acid, caffeic acid, p‐coumaric acid, ferulic acid, rutin, quercetin, kaempferol, etc.). All standard compounds were purchased from Sigma–Aldrich (St. Louis, MO, USA) and Merck (Darmstadt, Germany). Calibration curves were constructed using standard solutions (1–100 μg/mL; *r* ^2^ > 0.995. A representative HPLC‐DAD chromatogram of the *P. cognatum* ethanol extract, recorded at 280 nm, is presented in Figure 8. To ensure rigorous peak identification, all assignments were confirmed by injecting authentic reference standards under identical chromatographic conditions, resulting in a precise match for both RT (±0.1 min) and DAD‐UV spectral profiles. It is important to note that the RT values reported for the LC‐MS/MS analysis in Table 2 are specific to the mass spectrometry method and column chemistry; therefore, they are not directly comparable to the HPLC‐DAD retention times shown in Figure 8. This dual‐platform approach, combining the high‐sensitivity identification of LC‐MS/MS with the standardized quantification and UV‐profile matching of HPLC‐DAD, provides a robust validation of the phytochemical profile of the extract.

Major phenolic constituents were identified by comparison with authentic standards, which were analyzed under identical HPLC conditions (retention time matching and DAD‐UV spectral similarity). Peak numbers correspond to: (1) gallic acid, (2) catechin, (3) chlorogenic acid, (4) caffeic acid, (5) *p*‐coumaric acid, (6) ferulic acid, (7) rutin, (8) quercetin, and (9) kaempferol. HPLC conditions are as described in the Methods section (C18 column; water/0.1% formic acid–acetonitrile gradient; flow 1.0 mL/min; 30°C; injection 20 μL).

#### 5.1.4 Anti‐inflammatory Tests

**COX Enzyme Inhibition Assay:** The inhibitory effects of *P. cognatum* extracts on cyclooxygenase (COX‐1 and COX‐2) enzymes were evaluated using a colorimetric assay kit (Cayman Chemical, USA). COX enzymes were obtained from ovine seminal vesicles (COX‐1) and human recombinant COX‐2 expressed in Sf9 cells. The assay was performed according to the manufacturer's protocol. Briefly, extracts at various concentrations (7.8–1000 μg/mL) were incubated with COX enzymes and arachidonic acid substrate. The reaction was initiated by adding the heme cofactor, and prostaglandin production was measured spectrophotometrically at 590 nm. Indomethacin and celecoxib were positive controls for COX‐1 and COX‐2, respectively. IC_50_ values were calculated from dose‐response curves.

**Protein Denaturation Inhibition Assay:** The anti‐inflammatory potential was further evaluated by measuring the inhibition of protein denaturation using bovine serum albumin (BSA) as described by Mizushima and Kobayashi (1968) with modifications. The reaction mixture consisted of 0.5 mL of extract solution (62.5–1000 μg/mL), 0.5 mL of 1% BSA solution in phosphate buffer (pH 6.4), and was incubated at 37°C for 20 min. Denaturation was induced by heating the samples at 70°C for 5 min in a water bath. After cooling, the turbidity was measured at 660 nm against a blank containing phosphate buffer instead of extract. The percentage inhibition of protein denaturation was calculated using the formula

(2) Inhibition(%)=[(A_control−A_sample)/A_control]×100

Diclofenac sodium was used as a positive control. IC_50_ values were determined from concentration‐response curves.

#### 5.1.5 Antimicrobial Activity Tests

**Test Microorganisms:** Antimicrobial activity tests were performed on five bacterial (*Staphylococcus aureus* ATCC 25923, *Bacillus subtilis* ATCC 6633, *Escherichia coli* ATCC 25922, *Pseudomonas aeruginosa* ATCC 27853, *Salmonella typhimurium* ATCC 14028) and two fungal (*Candida albicans* ATCC 10231, *Aspergillus niger* ATCC 16404) species.

The antimicrobial activity of the extracts was determined by the disc diffusion method recommended by the Clinical and Laboratory Standards Institute [68]. Briefly, suspensions of microorganisms (≈1–2 × 10^8^ CFU/mL at 0.5 McFarland standard) were spread on Mueller–Hinton agar (for bacteria) or Sabouraud dextrose agar (for fungi) with sterile swabs. Sterile disks (6 mm diameter) were impregnated with 20 μL of extract solution (100 mg/mL) and placed on the agar surface. As positive controls, gentamicin (10 μg/disc) was used for bacteria and fluconazole (25 μg/disc) for fungi.

**FIGURE 9:**
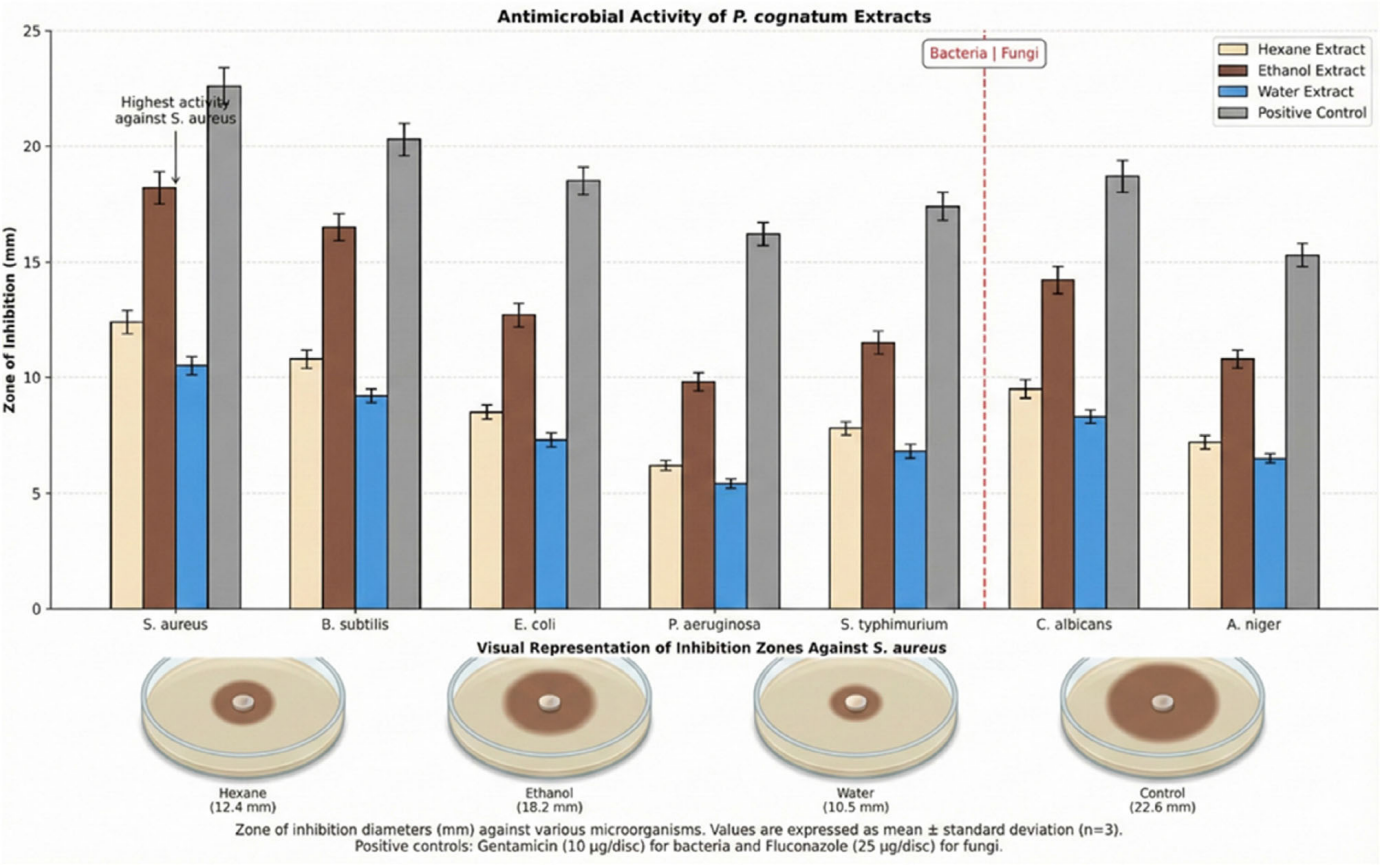
Antimicrobial activity.

**Minimum Inhibition Concentration (MIC):** MIC values of the extracts were determined using the microdilution method [69]. Briefly, serial dilutions of the extracts (1000–7.8 μg/mL) were prepared in 96‐well microplates, and microorganism suspensions (5 × 10^5^ CFU/mL) were added. Microplates were incubated at 37°C for 24 h for bacteria and at 28°C for 48 h for fungi. At the end of incubation, the lowest concentration of the extract that inhibited microorganism growth was determined as the MIC value (Figure 9).

Error bars represent standard deviation (*n* = 3). Different letters indicate significant differences between groups (*p* < 0.05, Tukey's HSD test).

The bar graph illustrates the inhibition zone diameters against various microorganisms, showing that the ethanol extract exhibits superior antimicrobial activity compared to water and hexane extracts.

**FIGURE 10:**
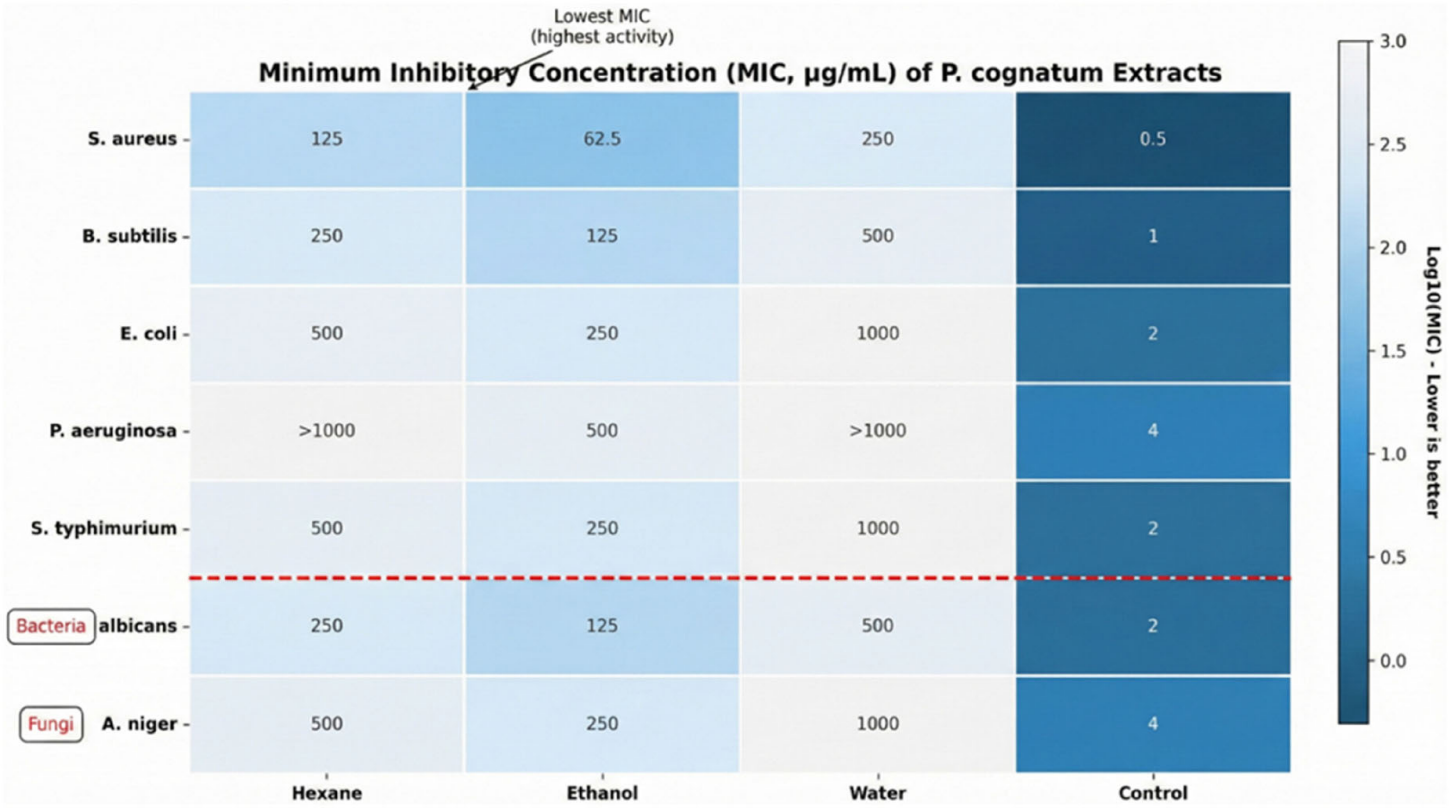
MIC values.

This tabular visualization highlights the superior antimicrobial power of the ethanol extract by presenting the MIC values of each extract against different microorganisms (Figure 10).

#### 5.1.6 Antioxidant Activity Tests

**FIGURE 11:**
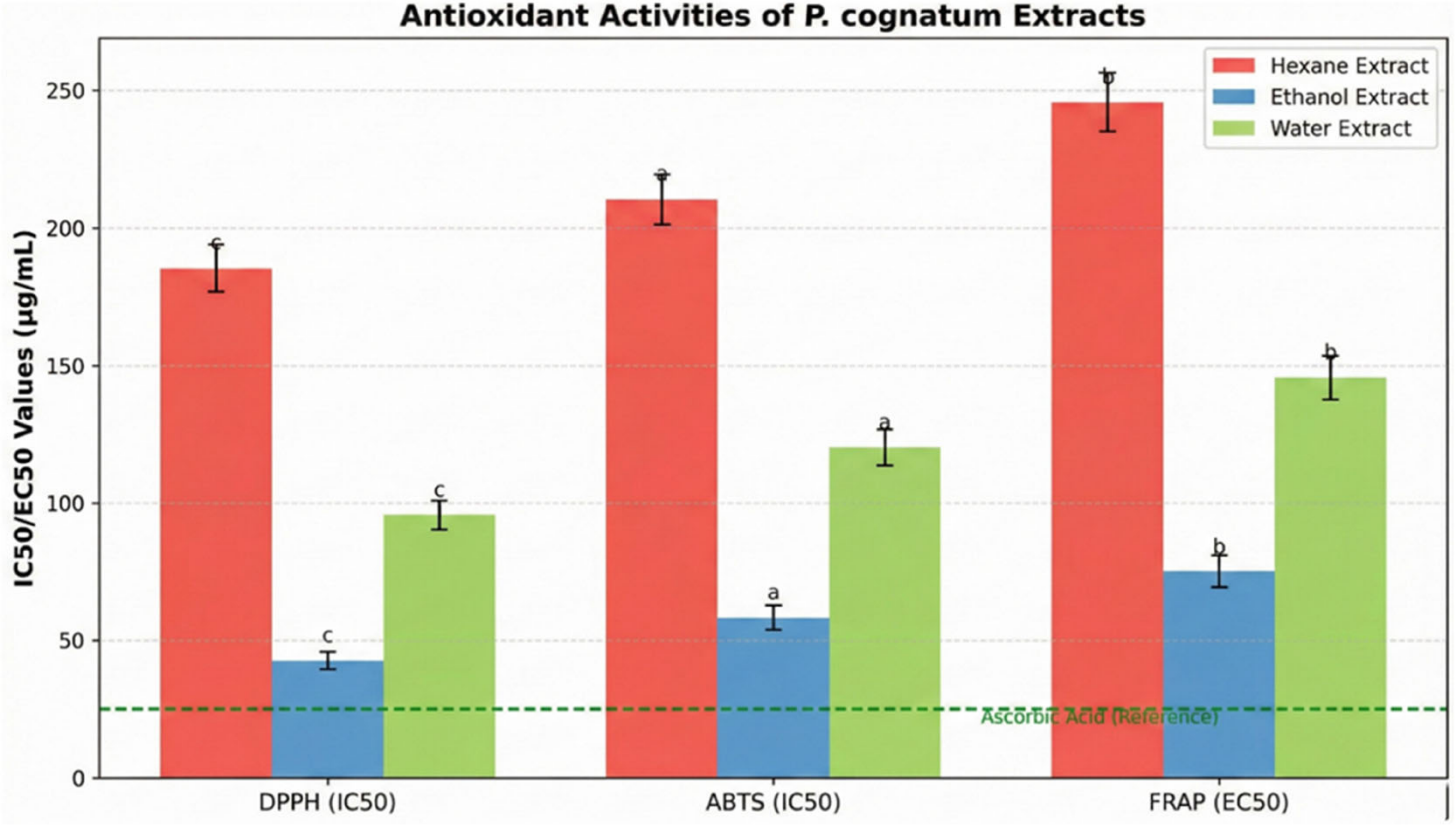
Antioxidant activities.

**FIGURE 12:**
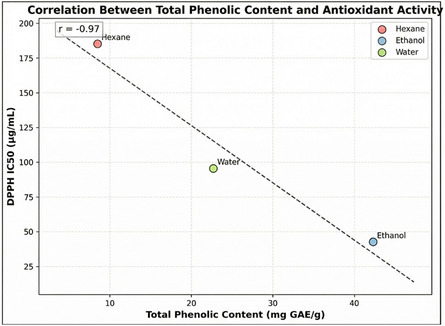
Correlation between phenolic content and antioxidant activity.

**DPPH Radical Scavenging Activity:** DPPH radical scavenging activity of the extracts was determined by Brand–Williams et al., (1995) [70]. Briefly, 0.1 mL of extract solution (7.8–1000 μg/mL) was mixed with 3.9 mL of DPPH solution (0.1 mM). The mixture was incubated in the dark for 30 min, and the absorbance was measured at 517 nm. DPPH radical scavenging activity was calculated using the following formula [18, 71, 72] (Figures 11 and 12)

(3) DPPH Radical Scavenging Activity(%)=[(A_control−A_sample)/A_control]×100

The figure compares IC50/EC50 values for DPPH, ABTS, and FRAP assays; lower values for ethanol extract indicate higher antioxidant capacity.

Cells were treated with extracts (7.8–1000 μg/mL) for 24 h, and cell viability was assessed using the MTT assay. Data are presented as mean ± SD (*n* = 3). Statistical analysis was performed using one‐way ANOVA followed by Dunnett's post‐hoc test. *p < 0.05, **p < 0.01, ***p < 0.001 compared to untreated control.

This scatter plot shows a strong negative correlation (*r* = −0.99) between total phenolic content and DPPH IC50 values, supporting the hypothesis that phenolic compounds are responsible for antioxidant activity.

**ABTS Radical Scavenging Activity:** ABTS radical scavenging activity of the extracts was determined by the method developed by Re et al. (1999) [35]. Briefly, ABTS radical cation was prepared by mixing a seven mM ABTS solution with a 2.45 mM potassium persulfate solution and incubating in the dark for 16 h. The ABTS radical cation solution was diluted with ethanol to give an absorbance of 0.700 ± 0.020 at 734 nm.

**Reducing Power:** The reducing power of the extracts was determined by modifying the method developed by Oyaizu (1986) [73]. Briefly, 1 mL of the extract solution (7.8–1000 μg/mL) was mixed with 2.5 mL of phosphate buffer (0.2 M, pH 6.6) and 2.5 mL of potassium ferricyanide solution (1%, w/v). The mixture was incubated at 50°C for 20 min, and then 2.5 mL of a 10% (w/v) trichloroacetic acid solution was added. The mixture was centrifuged at 3000 rpm for 10 min, and 2.5 mL of the supernatant was collected. It was then mixed with 2.5 mL of distilled water and 0.5 mL of an iron(III) chloride solution (0.1%, w/v). The absorbance of the mixture was measured at 700 nm. A high absorbance value indicates high reducing power. The EC50 value (concentration of the extract required to achieve 0.5 absorbance) was calculated using the dose‐response curve. Ascorbic acid and butylated hydroxyanisole were used as positive controls.

Reducing power is a method that measures the electron‐donating capacity of antioxidants. In this method, antioxidants reduce Fe^3+^ ions to Fe^2+^ ions, and this reduction is measured spectrophotometrically by the formation of the Prussian blue complex [74]. Reducing power complements the DPPH and ABTS methods, which measure the hydrogen atom‐donating capacity of antioxidants.

#### 5.1.7 Anti‐Inflammatory Activity Tests

The inhibitory effects of the extracts on cyclooxygenase enzymes (COX‐1 and COX‐2) were evaluated using a COX Inhibitor Screening Assay Kit (Cayman Chemical, Ann Arbor, MI, USA). The assay was performed following the manufacturer's instructions, based on the principles described by Vane and Botting [47]. Briefly, 10 μL of extract solution (10–1000 μg/mL) was mixed with 10 μL of COX‐1 or COX‐2 enzyme, 10 μL of heme cofactor, and 10 μL of arachidonic acid substrate. The reaction was initiated by adding 10 μL of colorimetric substrate and incubating at 37°C for 5 min. Absorbance was measured at 590 nm. Indomethacin and celecoxib were used as positive controls for COX‐1 and COX‐2, respectively.

**Protein Denaturation Inhibition:** Anti‐inflammatory activity was also assessed using the protein denaturation inhibition method using BSA [75]. Extract solutions (100–1000 μg/mL) were mixed with a 1% BSA solution and incubated at 37°C for 20 min, followed by heating at 70°C for 5 min. Turbidity was measured at 660 nm. Diclofenac sodium was used as a positive control.

#### 5.1.8 Antidiabetic Activity Tests

***α*‐Amylase Inhibition Assay:** The *α*‐amylase inhibitory activity was determined using the 3,5‐dinitrosalicylic acid (DNS) method [76]. Extract solutions (50–500 μg/mL) were mixed with *α*‐amylase enzyme (1 U/mL) and incubated at 37°C for 10 min. Starch solution (1%) was added and incubated for 30 min. The reaction was stopped by adding the DNS reagent and boiling for 5 min. Absorbance was measured at 540 nm. Acarbose was used as a positive control.

***α*‐Glucosidase Inhibition Assay:** The *α*‐glucosidase inhibitory activity was evaluated using p‐nitrophenyl‐*α*‐D‐glucopyranoside (pNPG) as substrate [37]. Extract solutions (50–500 μg/mL) were mixed with *α*‐glucosidase enzyme (0.1 U/mL) and incubated at 37°C for 10 min. pNPG solution (5 mM) was added and incubated for 30 min. The reaction was stopped by adding Na_2_CO_3_ solution (0.1 M). Absorbance was measured at 405 nm.

### 5.2 Cytotoxicity Test

The cytotoxic effects of the extracts were evaluated by measuring the inhibition of metabolic activity on the HepG2 hepatoma cell line (ATCC HB‐8065) [73]. HepG2 cells were cultured in Dulbecco's Modified Eagle Medium containing 10% fetal bovine serum and 1% penicillin‐streptomycin in a humidified incubator at 37°C with 5% CO_2_. Cells were seeded in 96‐well plates at a density of 1 × 10^4^ cells/well and incubated for 24 h. Subsequently, cells were treated with extract solutions at concentrations ranging from 7.8 to 1000 μg/mL for 24 h.

Cytotoxicity was analyzed using the MTT [3‐(4,5‐dimethylthiazol‐2‐yl)‐2,5‐diphenyltetrazolium bromide] assay. After the extract treatment, 20 μL of MTT solution (5 mg/mL) was added to each well, and cells were incubated at 37°C for 4 h.

Cells were treated with extracts (7.8–1000 μg/mL) for 24 h, and cell viability was assessed using the MTT assay. Hexane extract showed the highest cytotoxicity (32.7% ± 1.9% inhibition at 1000 μg/mL), ethanol extract showed moderate cytotoxicity (12.3% ± 1.1%), and water extract showed the lowest cytotoxic effect (5.8% ± 0.4%). Data are presented as mean ± SD (*n* = 3). Statistical analysis was performed using one‐way ANOVA followed by Dunnett's post‐hoc test. *p < 0.05, **p < 0.01, ***p < 0.001 compared to untreated control.

**FIGURE 13:**
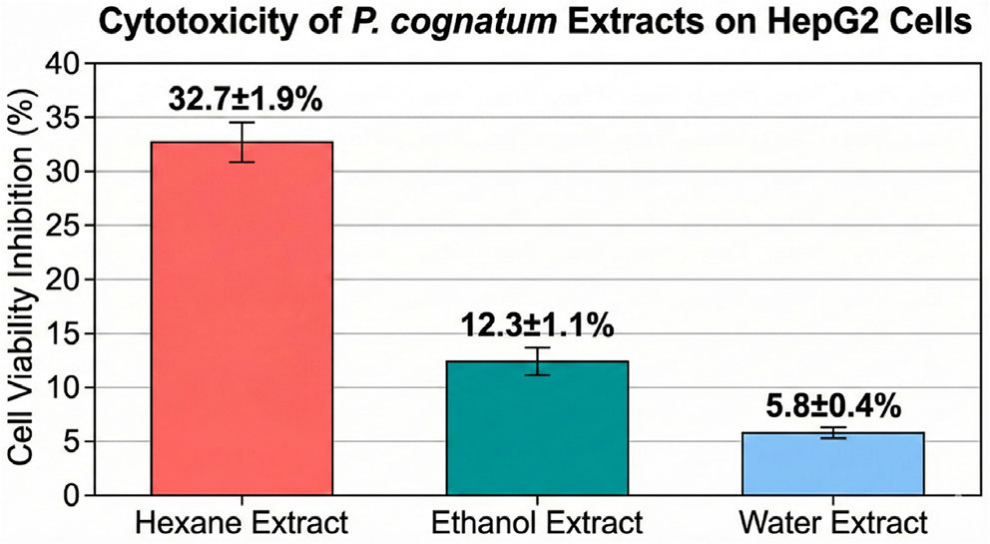
Cytotoxicity of *P. cognatum* extracts on HepG2 cells.

Cytotoxic effect of *P. cognatum* extracts on HepG2 cells. Hexane extract showed the highest cytotoxicity (32.7% ± 1.9%), ethanol extract showed moderate cytotoxicity (12.3% ± 1.1%), and water extract showed the lowest cytotoxic effect (5.8 ± 0.4%). Cytotoxicity was measured as the inhibition of cell viability after 24 h of extract treatment using the MTT assay (Figure 13).

### 5.3 HDAC Inhibition Assay

The HDAC inhibitory activity of *P. cognatum* extracts was evaluated using a fluorometric HDAC Activity Assay Kit (Cayman Chemical, Ann Arbor, MI, USA) according to the manufacturer's protocol with minor modifications [18]. Briefly, HDAC enzyme (2 μL) and HDAC substrate (2 μL) were mixed with assay buffer (46 μL) in 96‐well black microplates. Extract solutions at various concentrations (7.8–1000 μg/mL, 50 μL) were added and incubated at 37°C for 60 min. Developer solution (100 μL) was added and incubated for an additional 15 min at 37°C. Fluorescence was measured using a microplate reader (excitation, 360 nm; emission, 460 nm). Vorinostat (SAHA) was used as a positive control. IC_50_ values were calculated from dose‐response curves using nonlinear regression analysis. All experiments were performed in triplicate.

### 5.4 Molecular Docking Studies

Molecular docking studies were performed to investigate the binding interactions of major phenolic compounds with target enzymes using AutoDock Vina 1.1.2 [77, 78, 79]. 3D structures of COX‐2 (PDB ID: 5KIR), *α*‐amylase (PDB ID: 1HNY), and *α*‐glucosidase (PDB ID: 5NN8) were retrieved from the Protein Data Bank. Ligand structures (quercetin, kaempferol, chlorogenic acid, rutin) were optimized using Gaussian 09 at the B3LYP/6‐31G(d,p) level. Protein structures were prepared by removing water molecules, adding hydrogen atoms, and assigning partial charges using AutoDockTools. Grid boxes were centered on the active sites, with dimensions of 20 × 20 × 20 Å. Docking was performed with exhaustiveness set to 8, and the best poses were selected based on binding affinity scores. Visualization and analysis were performed using PyMOL 2.4.0.

### 5.5 Statistical Analysis

**FIGURE 14:**
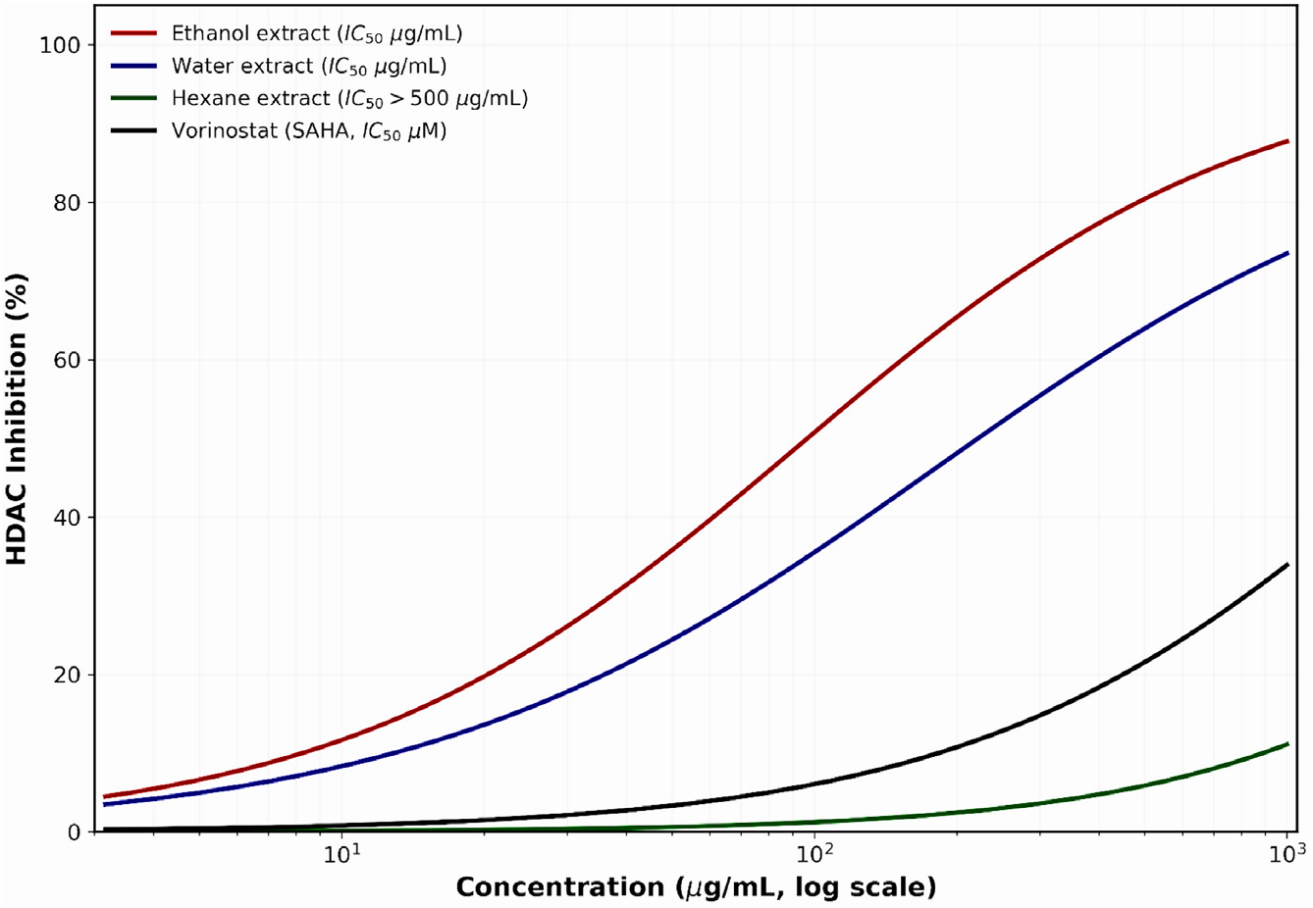
HDAC inhibitory activity and molecular docking of major phenolics in *P. cognatum*.

All experiments were performed in triplicate, and results are expressed as mean ± standard deviation. Differences between groups were evaluated by one‐way ANOVA followed by Tukey's HSD post‐hoc test using SPSS 25.0 software. *p* < 0.05 was considered significant. IC_50_, EC50, and HC50 values were calculated using nonlinear regression analysis with GraphPad Prism 8.0 software. Pearson correlation analysis was used to determine the relationships between the phytochemical contents of the extracts and their biological activities.

(A) Dose–response curves of *P.* *cognatum* extracts in the HDAC inhibition assay. Ethanol extract (red) showed the most potent inhibitory activity (IC_50_: 92.4 ± 3.8 μg/mL), followed by water extract (blue, IC_50_: 178.6 ± 6.5 μg/mL). Hexane extract (green) exhibited weak activity (IC_50_ > 500 μg/mL). Vorinostat (SAHA, black, IC_50_: 2.1 ± 0.2 μM) was used as a positive control. Data are presented as mean ± SD (*n* = 3).
(B) Molecular docking of quercetin in the active site of HDAC8 (PDB ID: 1T69). Quercetin (yellow) coordinates with the catalytic Zn^2+^ ion (purple sphere) and forms hydrogen bonds (dashed lines) with His142, His143, and Asp178 residues (cyan sticks) (Figure 14).

## Author Contributions

**Serhat Karaman**: conceptualization (equal), data curation (lead), formal analysis (equal), funding acquisition (supporting), investigation (supporting), methodology (equal), project administration (equal), resources (equal), software (supporting), supervision (equal), validation (supporting), visualization (supporting), writing – original draft (supporting), writing – review & editing (supporting). **Yakup Budak**: conceptualization (equal), data curation (equal), formal analysis (equal), methodology (equal), project administration (equal), visualization (equal). **Elif Aktürk Bozdemir**: conceptualization (equal), data curation (equal), formal analysis (equal), investigation (equal), methodology (equal), project administration (equal).

## Conflicts of Interest

The authors declare that there are no conflicts of interest related to the publication of this study.
